# Multilingual evaluation of interpretable biomarkers to represent language and speech patterns in Parkinson's disease

**DOI:** 10.3389/fneur.2023.1142642

**Published:** 2023-03-02

**Authors:** Anna Favaro, Laureano Moro-Velázquez, Ankur Butala, Chelsie Motley, Tianyu Cao, Robert David Stevens, Jesús Villalba, Najim Dehak

**Affiliations:** ^1^Department of Electrical and Computer Engineering, The Johns Hopkins University, Baltimore, MD, United States; ^2^Department of Neurology, The Johns Hopkins University, Baltimore, MD, United States; ^3^Department of Psychiatry and Behavioral Sciences, The Johns Hopkins University, Baltimore, MD, United States; ^4^Department of Anesthesiology and Critical Care, The Johns Hopkins University, Baltimore, MD, United States

**Keywords:** Parkinson's disease, interpretable biomarkers, speech, cognition, language, multilingual evaluation, correlation analysis, statistical analysis

## Abstract

Motor impairments are only one aspect of Parkinson's disease (PD), which also include cognitive and linguistic impairments. Speech-derived interpretable biomarkers may help clinicians diagnose PD at earlier stages and monitor the disorder's evolution over time. This study focuses on the multilingual evaluation of a composite array of biomarkers that facilitate PD evaluation from speech. Hypokinetic dysarthria, a motor speech disorder associated with PD, has been extensively analyzed in previously published studies on automatic PD evaluation, with a relative lack of inquiry into language and task variability. In this study, we explore certain acoustic, linguistic, and cognitive information encoded within the speech of several cohorts with PD. A total of 24 biomarkers were analyzed from American English, Italian, Castilian Spanish, Colombian Spanish, German, and Czech by conducting a statistical analysis to evaluate which biomarkers best differentiate people with PD from healthy participants. The study leverages conceptual *robustness* as a criterion in which a biomarker behaves the same, independent of the language. Hence, we propose a set of speech-based biomarkers that can effectively help evaluate PD while being language-independent. In short, the best acoustic and cognitive biomarkers permitting discrimination between experimental groups across languages were fundamental frequency standard deviation, pause time, pause percentage, silence duration, and speech rhythm standard deviation. Linguistic biomarkers representing the length of the narratives and the number of nouns and auxiliaries also provided discrimination between groups. Altogether, in addition to being significant, these biomarkers satisfied the robustness requirements.

## 1. Introduction

Parkinson's disease (PD) is a chronic progressive neurodegenerative disorder resulting from the gradual neuronal death in the substantia nigra, a region of dopamine neurotransmitter production. A hallmark of the condition is motor impairment, characterized by symptoms such as akinesia, bradykinesia, rigidity, resting tremor, and postural instability (1, 2). Approximately 1% of the population aged over 60 years in industrialized countries are affected by PD. As life expectancy increases, more than 9 million people are expected to be affected by 2030 (3). A *definite* PD diagnosis is established *via* autopsy (4) though clinical criteria based on patient history and physical examination are the practical norm (5). The average time needed to diagnose PD in a clinical setting can reach 2.9 years, with a diagnostic accuracy of ~80.6% (95% credible interval [CrI] 75.2–85.3%) (6, 7). In addition, tracking PD progression includes the adoption of subjective rating scales [e.g., (8)] that are considered to have low sensitivity and inter-rater reliability at the mild end of the symptom severity spectrum (9, 10). This is due, in part, to the variable and subtle nature of the early symptomatic presentation. Hence, new objective and non-invasive methodologies are required to speed up and support the current diagnostic techniques. In this respect, the early detection of PD, especially in its preclinical state, helps to slow the disorder's progression, diminishes its impact on patient's daily activities, and helps to identify therapeutic targets (11, 12). In addition, timely diagnosis can help alleviate the economic burden caused by PD to individuals, families, and the government, which is estimated to be ~*$* 51.9 billion annually in the U.S. (13).

The loss of dopamine neurons in the substantia nigra and the abnormal accumulation and aggregation of alpha-synuclein (α−Syn) in the form of Lewy bodies (LBs) is prototypical for PD (14). However, erstwhile Alzheimer's-specific pathological findings of neurofibrillary tangles and amyloid-β plaques are common (15, 16). The widespread neurodegeneration affects dopaminergic, cholinergic, noradrenergic, and (in late stages) serotonergic neurotransmission (17). Furthermore, the loss of ascending dopaminergic transmission has proximate effects on motor control *via* the basal ganglia and wider downstream effects on mood and cognition (18–20). Most immediately, the loss of dopaminergic input into the striatum and the consequent dysregulation of the basal ganglia result in the cardinal motor deficits that affect the anatomic subsystems of respiration, phonation, and articulation. However, the upstream, cortically-based control circuits underpinning language, articulation, phonation, and prosody are distributed (21, 22). These three subsystems govern speech–motor control. Up to 90% of subjects with PD, at some point, demonstrate the deterioration of phonation, articulation, prosody, and respiration, collectively reported as *hypokinetic dysarthria* (23). As speech production specifically requires the coordinated and precise activation of articulatory and laryngeal muscles, it is an excellent candidate for PD diagnosis. However, although PD is most often characterized by its motor symptoms, cognitive and linguistic manifestations are also prevalent. Cognitive changes in PD involve executive problems and are partially explained by a dopaminergic deficit, which causes a dysfunction of the cognitive pathways connecting the frontal lobes and basal ganglia (20). This impacts the executive function notably, including lexical retrieval and processing speed (24, 25). As other non-motor symptoms, recent studies found the incidence of major depressive disorder to be seen in ~17% of patients with PD, that of minor depression to be 22%, and that of dysthymia to be 13% (26).

Speech analysis can help identify patterns that cannot be distinguished from a clinical standpoint and thus help perform earlier diagnosis and monitor disease progression. To our knowledge, there are only a few reports analyzing speech-based biomarkers from a multilingual cohort of participants with PD. This gap in the literature can be explained by the inter-language variability that imposes considerable practical challenges for developing a unified speech assessment framework. In the past, Whitehill (27) found that the most deteriorated speech dimensions common in Cantonese-speaking and English-speaking persons affected by dysarthria (associated with PD) are related to voice quality, reduced pitch, loudness variation, and imprecise consonants. Kim and Choi (28) performed a descriptive study in which they analyzed the differences in acoustic vowel space (AVS) of Korean- and American-English-speaking subjects with PD. No differences in the articulation rate were observed. Rusz et al. (29) performed a speech analysis of Czech, English, German, French, and Italian speakers in the early phase of PD, recording a cohort of 448 participants. Monopitch, length of pauses, and imprecise consonants significantly differentiated subjects based on speech into control (CN) and PD groups. Kovac et al. (30) focused on the multilingual analysis of a set of acoustic speech biomarkers intended to support PD diagnosis. They used speech recordings from 214 Czech-speaking subjects, 29 American English-speaking subjects, 115 Israeli-speaking subjects, 100 Colombian Spanish-speaking subjects, and 48 Italian-speaking subjects. The biomarkers quantifying the prominence of the second formant, monopitch, and the number of pauses detected during text reading showed the best results in discriminating participants with PD from CN participants in the statistical analysis.

Most of the previous studies have different limitations. Some only analyzed acoustic descriptors, which limit the characterization of PD to a unique domain, discarding cognitive and linguistic information that can help phenotype the disorder. Others only considered a single language task (e.g., sustained vowel phonation and diadochokinetic task) or a few languages simultaneously. It follows that the multilingual analysis of PD requires expanding the analysis to a broader set of languages, focusing on a more comprehensive set of descriptors belonging to different domains and collecting speech samples from different tasks to deliver an exhaustive characterization of the disorder. This study performs a multilingual analysis of speech-based interpretable biomarkers encoding acoustic, linguistic, and cognitive-related information aimed at advancing the understanding of the specificity and commonalities of PD-pathology in acoustic, linguistic, and cognitive patterns, which are *universal*. The biomarkers presented in this study can be obtained automatically, without human supervision and, presumably, without human bias. Moreover, they have the advantage of encoding meaningful information that can be easily understood by clinicians. Another important contribution of the current study is the data collection. As detailed in Section 2.1.1, in this study, the authors collected speech recordings from American English native speakers with and without neurological disorders (NDs) at Johns Hopkins Medicine (JHM).

## 2. Materials and methods

### 2.1. Data sets

We assessed the effectiveness of different biomarkers by performing a statistical analysis to evaluate the power of the biomarkers in differentiating between the speech of participants with PD and CN participants across earlier published data sets against a novel corpus (i.e., NeuroLogical Signals). We also conducted a correlation analysis between the same biomarkers and clinical scores (see Section 2.4). A total of six different data sets were included: NeuroLogical Signals (NLS) (31), Neurovoz (32), Italian Parkinson's Voice and Speech (ItalianPVS) (33), GITA (34), GermanPD (35), and CzechPD (36). Only these six data sets were considered because they were the only ones to which we had or received access. These six data sets vary in demographics, collection procedures, and sizes and are described in the following subsections. From each data set, we analyzed three types of tasks (when available): a spontaneous speech (SS) sample (e.g., monologue and picture description), a reading passage (RP), and text-dependent utterances (TDUs) (short sentences). Table 1 summarizes the types of tasks employed in this study and the data sets in which these tasks are available. In the Supplementary material, a table containing information (when available) about medication state, time since diagnosis, time since medication, microphone typology, gender distribution, age range, and mask presence during the recording session is reported for each data set. Altogether, since these variables can influence speech emission, they will be considered in detail when assessing the robustness of the biomarkers (see Section 3).

**Table 1:** Typologies of tasks analyzed with the corresponding number (#) of samples available in each data set.

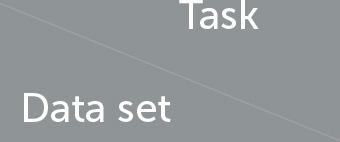	**SS**	**RP**	**TDU**
NLS	1	2	0
Neurovoz	1	0	6
GermanPD	1	1	5
CzechPD	1	1	0
GITA	1	1	6
ItalianPVS	0	2	10

SS, spontaneous speech; RP, read passage; TDU, text dependent utterance.

#### 2.1.1. NeuroLogical signals

NeuroLogical Signals (NLS) are a form of data set collected by the authors of this study at JHM. It contains spoken responses to several tasks of participants whose native language is American English. All participants were either categorized as having a ND or were categorized as CN. All of them were seen at the Johns Hopkins Health System and gave informed consent. The Johns Hopkins Medical Institutional Review Board approved data collection. Expert neurologists at JHM diagnosed and treated participants with NDs. As this study was performed during the COVID-19 pandemic, all participants wore the same type of surgical mask during recordings. The participants with PD continued their usual (individualized) pharmacological treatment and took dopaminergic medication (i.e., L-Dopa) between 1 and 5 h before the recording session. Speech signals were recorded with the help of a computer and a microphone at 24 kHz with a resolution of 16 bits in acoustically and visually controlled and consistent conditions. Inclusion criteria for the participants with NDs included being English native speakers and literate. A trained research assistant gave participants specific instructions on how to complete each task before the start of each recording session. In this study, we considered 23 participants with clinically established PD, and we created a CN group of 27 participants with matching age of that of the PD group. None of the participants in the CN group has a history of symptoms related to PD or any other NDs. Table 2 contains the demographic and disease severity statistics of the two experimental groups. The subset of tasks analyzed from this corpus consists of a SS task and two RP tasks. The SS task is represented by a Cookie Theft Picture description task (37), in which participants are required to describe the Cookie Theft Picture from the Boston Diagnostic Aphasia Examination. The protocol set a limit of 60 *s* on the execution of this task. The speech transcripts of the two RPs are reported in the Supplementary material.

**Table 2:** Demographic and disease severity statistics of NLS, GermanPD, and ItalianPVS data sets.

**Dataset**	**NLS**				**German PD**				**ItalianPVS**			
**Gender**	**Female**		**Male**		**Female**		**Male**		**Female**		**Male**	
**Group**	**PD**	**CN**	**PD**	**CN**	**PD**	**CN**	**PD**	**CN**	**PD**	**CN**	**PD**	**CN**
Number of subjects	9	16	14	11	41	44	47	44	9	12	19	10
Age, average	68.00	63.31	66.62	66.09	67.23 (9.7)	62.6 (15.2)	66.7 (8.7)	63.8 (12.7)	64.3 (12.2)	65.3 (4.1)	68.6 (6.4)	69.3 (5.6)
Age range	50–75	26–83	55–79	41–79	27–84	28–85	44–82	26–83	40–80	60–72	50–77	60–77
UPDRS-III*, average (SD)	25.33 (9.72)	–	27.71 (13.63)	–	23.3 (12.0)	–	22.1 (9.9)	–	–	–	–	–
UPDRS-III.I average (SD)	0.88 (0.56)	–	0.83 (0.68)	–	–	–	–	–	1.10 (1.3)	–	1.00 (1.00)	–
Hoehn and Yahr* scale average (SD)	2.37 (0.41)	–	2.23 (0.37)	–	2.61 (0.83)	–	2.59 (0.60)	–	–	–	–	–
Years since diagnosis (SD)	–	–	–	–	6.47 (5.83)	–	6.59 (4.93)	–	–	–	–	–

Ages are expressed in years. *NLS contains the global values of Movement Disorder Society UPDRS part III (i.e., movement examination); GermanPD contains the global values of UPDRS part III (i.e., movement examination). The ItalianPVS corpus only contains UPDRS-III.I (i.e., speech examination). The Hoehn and Yahr* scale is available only for NLS and GermanPD, but not for ItalianPVS.

#### 2.1.2. Neurovoz

Neurovoz is a data set that contains recordings from 47 CN and 44 participants with PD whose native language is Castilian Spanish. Table 3 contains the demographic and disease severity statistics of the two experimental groups. The samples were retrieved by the Bioengineering and Optoelectronics Group at Universidad Politecnica de Madrid and by the Otorhinolaryngology and Neurology Departments of the Gregorio Marañón hospital (Madrid, Spain). The ethics committee of Hospital General Universitario Gregorio Marañón approved the experimental protocols and methods according to the Helsinki Declaration (38). All participants involved in the study signed informed consent. The neurological state of participants with PD was assessed by a neurologist before the recording session. Differently, a survey was administered to evaluate the neurological condition of CN participants. In addition, a speech therapist assessed PD and CN participants' voices perceptually and through a survey. Participants who reported either neurological or organic pathologies (other than PD) were discarded. The participants with PD were under pharmacological treatment and took dopaminergic medication between 2 and 5 h before the recording session. Speech signals were collected in a room with controlled acoustic characteristics using an AKG C420 headset microphone coupled to a preamplifier with phantom power. The signal from the preamplifier was directed to a Soundblaster Live 24 bits sound card connected to a personal computer supplied with the software Medivoz (39). Recordings were sampled at 44.1 kHz with a resolution of 16 bits. The subset of tasks employed in this study contains recordings from six TDUs and running speech from the description of a picture (SS task). The protocol did not impose a time limit on the execution of the SS task. With respect to the TDUs, subjects were not required to read these sentences from a text document, but they listened to them from a recording of a standard speaker and repeated them out loud. This procedure was applied to reduce the noise of paper during recording, reading mistakes caused by visual impairments, and the cognitive load of the reading process. All tasks were performed at a comfortable speech sound pressure level (SPL). The speech transcripts of the TDUs are reported in the Supplementary material.

**Table 3:** Demographic and disease severity statistics of Neurovoz, GITA, and CzechPD data sets.

**Data set**	**Neurovoz**				**GITA**				**CzechPD**	
**Gender**	**Female**		**Male**		**Female**		**Male**		**Male**	
**Group**	**PD**	**CN**	**PD**	**CN**	**PD**	**CN**	**PD**	**CN**	**PD**	**CN**
Number of subjects	18	18	29	14	25	25	25	25	20	16
Age, average (SD)	70.9 (8.4)	68.4 (6.0)	71.9 (12.3)	66.6 (6.4)	60.7 (7.3)	61.4 (7.0)	61.5 (11.6)	60.5 (11.6)	61 (11.7)	61.8 (12.9)
Age range	59–86	58–83	41–88	55–77	49–75	49–76	33–81	31–86	34–83	36–80
UPDRS-III*, average (SD)	16.9 (11.5)	–	19.6 (11.8)	–	37.5 (14.0)	–	37.7 (22.0)	–	17.9 (7.1)	–
Hoehn and Yahr scale average (SD)	2.30 (0.68)	–	2.30 (0.80)	–	2.28 (0.53)	–	2.34 (0.53)	–	2.17 (0.45)	–
Years since diagnosis (SD)	6.4 (6.4)	–	7.6 (4.7)	–	12.6 (11.5)	–	8.9 (5.9)	–	2.4 (1.6)	–

Ages are expressed in years. *The Neurovoz corpus only contains UPDRS-III, i.e., motor examination; GITA contains the global values of Movement Disorder Society UPDRS; CzechPD contains the global values of UPDRS.

#### 2.1.3. GITA

GITA is a data set collected by Universidad de Antioquia in Medellín (Colombia). It contains a variety of speech tasks from 50 subjects with PD and 50 age- and sex-matched subjects in CN whose native language is Colombian Spanish. Table 3 contains the demographic and disease severity statistics of the two experimental groups. The data collection was performed in compliance with the Helsinki Declaration and was approved by the ethics committee of the Clínica Noel in Medellín. All participants signed informed consent. Patients were diagnosed with PD by neurologist experts. Speech samples were recorded with the patients in ON-state with dopaminergic medication (i.e., not more than 3 h after medication). None of the CN participants reported symptoms associated with PD or other NDs. Speech signals were collected in a quiet room using a dynamic omnidirectional microphone (Shure, SM63L). Recordings were sampled at 44.1 kHz with a resolution of 16 bits. A total of three types of speech tasks were used in this study: a SS task (i.e., monologue) where participants describe what they commonly do on a normal day (i.e., when they wake up or what kind of activities they do), six TDUs, and one phonetically balanced RP. The protocol did not impose a time limit on the execution of the SS task. SS samples last approximately for 44 *s*. The speech transcripts of the RP and TDU tasks are reported in the Supplementary material.

#### 2.1.4. GermanPD

GermanPD is a data set collected in the hospital of Bochum (Germany). It contains speech recordings from 88 PD and 88 CN participants whose native language is German. Table 2 contains the demographic and disease severity statistics of the two experimental groups. The study was approved by the ethics committee of the Ruhr-University Bochum. All participants signed informed consent. All participants with PD were in stable ON-state with dopaminergic medication unchanged since at least 4 weeks before the examination. Each participant with PD underwent a neurological examination before performing the speech task. To ensure the ON-state, speech and motor examinations were performed 60–90 min after the morning dose of L-Dopa. Speech samples were collected in a quiet room using commercial audio software (Steinberg WaveLab; Steinberg, Hamburg, Germany) and a headset microphone (Plantronics Audio 550 DSP; Plantronics Inc., Santa Cruz, CA), located ~5 cm from the participant's mouth. Recordings were sampled at 16 kHz with a resolution of 16 bits. The subset of tasks analyzed in this study contains recordings from 10 TDUs, one RP composed of 81 words, and a SS task (i.e., monologue). The protocol did not impose a time limit on the execution of the SS task. SS samples last approximately for 33 *s*. The speech transcripts of the TDUs are reported in the Supplementary material.

#### 2.1.5. Italian Parkinson's voice and speech

The ItalianPVS corpus^1^ contains recordings from 22 elderly CNs, 28 young CNs, and 28 participants with PD. Table 2 contains the demographic and disease severity statistics of the two experimental groups. The recordings employed in this study are publicly available. The information relevant to the participant's informed consent is detailed in their reference articles or dissemination platforms, with all cited in this article. None of the participants with PD reported any speech or language disorders unrelated to their PD symptoms before this study. All participants with PD were receiving antiparkinsonian treatment before the recording session. Each recording session occurred under a controlled environment, considering factors including room temperature, microphone distance, time of day, and conversing with the subject to warm up their vocal tract muscles. Speech samples were collected in a quiet, echo-free room, keeping a distance from the microphone of 15–25 cm. Recordings were sampled at 16 kHz. The subset of tasks employed in this study contains recordings from 10 phonetically balanced TDUs and one phonetically balanced RP. Before starting the reading tasks, a specialist introduced the participants to the task to be performed. Both the short sentences and the RP were read from a printed sheet. The speech transcripts of the RP and TDU tasks are reported in the Supplementary material.

#### 2.1.6. CzechPD

CzechPD is a data set collected in the General University Hospital in Prague (Czech Republic). It contains speech recordings from 20 newly diagnosed and untreated participants with PD and 15 CN whose native language is Czech. All the participants are men. Table 3 contains the demographic and disease severity statistics of the two experimental groups. The data collection was performed in compliance with the Helsinki Declaration and was approved by the ethics committee of the General University Hospital in Prague. All participants signed informed consent. None of the participants with PD received antiparkinsonian treatment before the recording session. A neurologist specializing in movement disorders established a PD diagnosis and performed the clinical evaluation. Speech samples were collected in a quiet room with an external condenser microphone located ~15 cm from the participant's mouth. Recordings were sampled at 48 kHz with a resolution of 16 bits. Every participant was recorded in a unique session with the speech-language pathologist. No time limits were imposed during the recording. Before starting the session, participants were familiarized with the tasks and procedure. In each recording, the participants performed various speaking tasks as a part of the larger protocol. The subset of tasks analyzed in this study contained recordings from a RP task and a SS task (i.e., monologue), where participants were required to speak about what they did that day or the previous week, their interests, job, or family. SS samples last approximately for 90 s. The speech transcripts of the RP task are reported in the Supplementary material.

### 2.2. Data pre-processing

All recordings were resampled at 16 kHz as required by the algorithms employed for the biomarker extraction (see Section 2.3). The resampling was performed using the sound processing program SoX^2^. We also applied the EBU R128 loudness normalization procedure using the Python library *ffmpeg-normalize*^3^. This type of normalization leads to a more uniform loudness level compared to simple peak-based normalization. Normalized audios were used to extract intensity-related biomarkers (see Section 2.3.1). Moreover, to perform the analysis of the TDUs, we concatenated the recordings speaker-wise into a single recording. By doing this, we trimmed silences at the end and beginning of each recording. All the recordings in the NLS data set were supervised to ensure they had appropriate quality. Criteria for acceptable quality included minimal background noise and a task-related response. When the recordings contained speech from the investigator at the beginning and end, we trimmed the recordings. All recordings from the SS task were automatically transcribed using OpenAI's Whisper^4^ (40), that is an Automatic Speech Recognition (ASR) system trained for 680,000 h of multilingual and multitask supervised data collected from the web.

### 2.3. Biomarker extraction

To provide an exhaustive characterization of the speech of participants with PD, we configured a set of interpretable biomarkers, while some are already proposed in our earlier works (31, 41), that encodes cognitive, acoustic, and linguistic information. Table 4 reports the description, the dysfunction connected to the occurrence, and the expected behavior for each biomarker. The expected behavior of a given biomarker in the PD population is grounded in the clinical literature documenting the common language and speech dysfunctions occurring in PD and, more generally, in NDs. In this regard, an ideal cross-lingual PD biomarker is a biomarker that displays a homogeneous behavior cross-lingually. The values of the extracted biomarkers were used to perform a non-parametric statistical analysis to identify which of the biomarkers significantly differed between the PD group and the CN group and to analyze commonalities (if any) in the behavior of the biomarkers across languages.

**Table 4:** Extracted acoustic, linguistic, and cognitive biomarkers.

**Task**	**Name**	**Biomarker description**	**Dysfunction**	**EB**
**Acoustic**				
All	F0STD [Hz]	Pitch variability defined as	Monopitch	↓
		the standard deviation of F0 contours		
All	F1STD [Hz]	Standard deviation of the first formant	Rigidity of tongue and jaw	↓
All	INTSTD [dB]	Speech loudness variability defined as the	Monoloudness	↓
		standard deviation of the intensity countour		
SS	SPTIME [s]	Net speech time relative to total speech time	Lower amount of speech time	↓
			in spontaneous production	
RP	SPTIME [s]	Net speech time relative to total speech time	Higher amount of speech time	↑
			due reading difficulties	
All	SILTIME [s]	Total amount of silent time in a recording	Higher amount of silent time	↑
All	SILPERC [%]	Total silent time divided by the total speech time	Higher amount of silent time	↑
		expressed in percentage		
All	SILSPPRAT [%]	Pause-speech ratio derived as the total amount of	Higher pause-speech ratio	↑
		silent time over the total amount of speech time		
All	SILDUR [s]	Mean length duration of speech pauses	Longer duration of silences	↑
All	SILVAR [s]	Median absolute deviation of speech pauses duration	Higher variability of silence time	↑
**Linguistic**				
SS	WORDCNT	Total word count after function words removal	Shorter narratives	↓
SS	WORDLEN	Mean word length computed in terms of	Difficulties in word completion/	↓
		the # of word characters after function words removal	adoption of less complex vocabulary	
SS	SENTCNT	Count of the # of sentences used in elicited narratives	Shorter/poorer linguistic production	↓
SS	SENTLEN	Sentence length defined in terms of the total number	Shorter and simpler narratives	↓
		of words		
SS	NOUNCNT	# of nouns	Selective impairment in object naming	↓
SS	VERBCNT	# of verbs	Selective impairment in action verbs	↓
SS	ADJCNT	# of adjectives	Reduced use of adjectives	↓
SS	ADVCNT	# of adverbs	Reduced use of adverbs	↓
SS	NUMCNT	# of numerals	Reduced use of numerals	↓
SS	AUXCNT	# of auxiliaries	Reduced use of auxiliaries	↓
SS	NPCNT	# of noun phrases	Reduced number of noun phrases	↓
SS	VPCNT	# of verb phrases	Reduced number of verb phrases	↓
SS	PPCNT	# of prepositional phrases	Reduced number of prepositional phrases	↓
**Cognitive**				
SS	IU	# informational units defined as	Selective impairment in action naming	↓
		as # of salient events occurring in a picture		
SS	RHYSTD	Speech rhythm defined as standard deviation of	Static speech rhythm	↓
		of word starting timestamps		
RP	RHYSTD	Speech rhythm defined as standard deviation of	Irregular speech rhythm	↑
		of word starting timestamps		

For each biomarker, we report the task from which the biomarker has been extracted, the biomarker name, the biomarker description, the specific dysfunction behind the biomarker, and the expected behavior (EB) occurring in PD subjects. #, number.

↑ means increasing, ↓ means decreasing.

#### 2.3.1. Acoustic biomarkers

Prosody is the systematic arrangement of various linguistic units into a single utterance or group of utterances, which occurs in speech production. Its realization encompasses both segmental and suprasegmental features of speech and aims at conveying linguistic and non-linguistic information (42). Prosody is one of the most deteriorated speech dimensions in hypokinetic dysarthria. To model the difficulties of subjects with PD in modulating pitch and loudness, we computed biomarkers encoding pitch variation that is defined as the standard deviation of fundamental frequency (F0) contours (F0STD), and loudness variation that is defined as the standard deviation of intensity contours (INTSTD). INSTD measures the ability of a person to keep the stability of air pressure produced by the lung. Moreover, to represent the rigidity of the tongue and jaw, we computed the standard deviation of the first formant (F1STD). To compute F0STD and F1STD, we used Disvoice^5^, that is a Python library designed to extract phonological, prosodic, articulatory, and glottal biomarkers from speech. To compute the fundamental frequency, this library uses the PRAAT algorithm^6^. The intensity I (dB) of an input signal was calculated using Parselmouth^7^, that is a Python library for the Praat software^8^. During both the RP and SS tasks, we hypothesized that participants affected by PD would display a reduced ability to modulate pitch and loudness. Moreover, we used DigiPsychProsody^9^, that is a Python library to compute biomarkers related to pauses such as total speech time (SPTIME), total pause time (SILTIME), percentage pause time (SILPERC), mean pause duration (SILDUR), silence to speech ratio (SILSPRAT), and pause variability (SILVAR). This library uses the WebRTC Voice Activity Detector^10^. We expected that subjects with PD would exhibit a higher SILDUR, SILTIME, SILPERC, SILSPRAT, and SILVAR due to the difficulties introduced by the reading (43) and by the construction of coherent narratives (44), respectively. Moreover, we expected that participants with PD would deliver shorter narratives than the CN group, as previously shown by Murray (45), resulting in lower SPTIME in the SS task. On the contrary, in the RP task, we expected that participants with PD would report a higher SPTIME due to word repetitions and self-corrections (46).

#### 2.3.2. Linguistic biomarkers

Individuals with early stages of PD report selective impairments in syntax and semantics, especially in action-verbs and action semantics, with relative preservation of noun processing (47). To analyze the syntactic constructions of subjects with PD, we calculated the frequency of occurrence of different parts of speech (POS), such as nouns (NOUNCNT), verbs (VERBCNT), adjectives (ADJCNT), adverbs (ADVCNT), numerals (NUMCNT), and auxiliaries (AUXCNT) during spontaneous production. We also measured the syntactic complexity in terms of the number of words (WORDCNT), average word length in characters (WORDLEN), number of sentences (SENTCNT), average sentence length in words (SENTLEN), number of noun phrases (NPCNT), number of verb phrases (VPCNT), and number of prepositional phrases (PPCNT). The WORDCNT biomarker was extracted after removing the function words (e.g., articles, pronouns, and prepositions) from the transcripts. We used the pre-trained language model pipeline for English, German, and Spanish available on Spacy to extract these biomarkers^11^. Since there is no available pipeline for Czech to perform POS tagging and syntactic analysis, we did not conduct the linguistic analysis on the CzechPD. The experimental hypothesis behind this analysis is grounded in the clinical literature: subjects with PD may deliver narratives with a lower level of syntactic complexity and elaboration, namely, their linguistic production may exhibit a lower number of syntactic phrases and categories (e.g., NOUNCNT and VPCNT) than CN participants (48–50).

#### 2.3.3. Cognitive biomarkers

Real-time adjustments of speech are crucial for effective communication. Therefore, speech deficits may contribute to the common observation that the speech of individuals with PD is more static, less empathetic, and semantically clear (51). In our analysis, we modeled the regularity of speech rhythm (RHYSTD) in terms of the occurrence of the individual words in time, namely, we measured the time between the starting points of consequent words and computed the standard deviation of these measurements for each recording. To derive the starting point of each word, we computed word alignment using WhisperX (52), that is a Python repository that improved the accuracy of the timestamps of the Whisper model *via* forced alignment with phoneme-based ASR models [e.g., wav2vec2.0; (53)]. The phoneme ASR alignment model is language-specific and is automatically imported from torchaudio pipelines^12^. Since there is no available tested phoneme ASR model for Czech, we did not extract this biomarker from CzechPD. We hypothesized that subjects with PD would show a lower RHYSTD (i.e., more static rhythm) than CN in the SS task, while they would show higher RHYSTD (i.e., more irregular rhythm) than CN in the reading tasks, as studied by Skodda et al. (54).

In addition, previous studies reported that subjects with PD (with and without dementia) demonstrated reduced discourse informativeness, which reflects disruptions to both conceptual and lexical discourse processes (55). We examined the informativeness of the spoken language samples in terms of the number of correct informational units (IU). According to Nicholas and Brookshire (56), IUs are *words that are intelligible in context, accurate in relation to the picture(s) or topic, and relevant to and informative about the content of the picture(s) or the topic*. In our analysis, we used the speech transcripts of the recordings collected during the Cookie Theft Picture (CTP) description task contained in NLS and during the picture description task contained in Neurovoz. We considered that as IUs, the salient events displayed in the picture presented as a stimulus to elicit the narrative. To identify the salient IUs for the CTP task, we used the published checklists for the CTP (57, 58). Thus, IUs were represented by the verbs like *washing, drying, stealing, overflowing, trying to help, falling, wobbling, hanging, ignoring, reaching up, asking for cookie, laughing*, and *standing*. On the contrary, in the picture description in Neurovoz, we used the verbs *barrer, lavar, pesar*, and *ducharse*. We hypothesized that subjects with PD would report a significantly lower number of IUs than CNs, as reported in the literature (45).

### 2.4. Statistical and correlation analysis

As we observed that not all the biomarkers were normally distributed, we used the Kruskal–Wallis *H*-test to conduct pair-wise statistical tests to determine if there were significant differences between the distributions of PD and CN for each biomarker. The analysis was performed in each language separately. The Kruskal–Wallis test (59) is a non-parametric test whose null hypothesis is that the median ranks of the groups are the same. To control the False Discovery Rate (FDR), we applied the Benjamini–Hochberg correction to each pair-wise comparison for each family of biomarkers (60). As family-wise error rate, we set α to 0.05. To perform the pair-wise Kruskal–Wallis *H*-tests, we used *scipy.stats.kruskal*^13^ library in Python, whereas to perform the Benjamini–Hochberg correction, we used *statsmodels.stats.multitest.fdrcorrection*^14^ with a default method. As the second level of analysis, the correlations of the biomarker values with motor symptoms (i.e., UPDRS-III, UPDRS-III.I, and H&Y scale) were measured^15^. Spearman's rank-correlation coefficients give us information about the negative or positive correlation of the biomarker with the clinical scores. To perform the correlation analysis, we used *scipy.stats.spearmanr* library in Python. As in the statistical analysis, we applied Benjamini–Hochberg correction to each pair-wise comparison for each family of biomarkers. As a normalization procedure before the correlation analysis, standardization was applied to each biomarker separately.

### 2.5. Biomarker robustness

We determined the robustness of each given biomarker from the statistical analysis results. We considered that a biomarker is *robust* if:

- The difference between the medians of the PD and CN distributions is significant in at least *two* data sets.
- It displays exactly the same expected behavior in the data sets where it results to be significant.
- The significant correlation of the biomarker with the clinical scores follows the expected behavior of the biomarker.

The expected cross-lingual behavior of each biomarker is reported in Table 4. Similar criteria for robustness were previously identified by Kovac et al. (30). However, the authors in that study considered robust even biomarkers that only differentiate the PD group from the CN group in one data set. These criteria for robustness have been applied also considering external factors (e.g., gender and disease severity) that could have influenced the trends of the biomarkers in the different languages.

## 3. Results and discussion

Tables 5, 6 report the pairwise Kruskal–Wallis *H*-test results for the acoustic, linguistic, and cognitive biomarkers that are statistically significant (*p* < 0.05). For each of the significant biomarkers, we report the *H*-statistics, the corresponding *p*-value, the eta-squared effect size (η^2^) based on the *H*-statistics, and the area under the ROC curve (AUROC). The AUROC of a biomarker can be used as a criterion to measure biomarker's discriminative properties. Moreover, we report the observed behavior (OB) of the biomarker that represents the direction in which the median value of the PD biomarker behaved compared to that of the CN group. Table 7 reports the significant correlations between the biomarkers and the different clinical scores, namely, UPDRS-III (motor assessment), UPDRS-III part I (speech assessment), and the Hoen and Yahr (H&Y) scale. In both the statistical and correlation analysis, Benjamini–Hochberg correction was applied to control for FDR. According to the Kruskal–Wallis *H*-test, among the 24 types of biomarkers that have been analyzed, 15 were significant on GermanPD, 13 on Neurovoz, nine on ItalianPVS, nine on NLS, seven on CzechPD, and four on GITA. Although 21 biomarkers were significant in at least one data set, only 13 strictly satisfied the robustness conditions. In summary, the language-robust biomarkers identified in our analysis had a lower amount of speech time in spontaneous production (SPTIME), a monotonic pitch (F0STD), and an increased amount of pauses and hesitations (SILTIME, SILPERC, SILVAR, SILSPRAT, and SILDUR) across tasks. From the linguistic analysis, it emerged that participants with PD delivered shorter narratives (WORDCNT and SENTCNT) and reduced the use of nouns, auxiliaries, and noun phrases (NOUNCNT, AUXCNT, and NPs). In addition, they reported a more static speech rhythm (RHYSTD) during spontaneous production. In Sections 3.1 and 3.2, these findings are presented in detail and compared to prior-related research better to understand the novelty of the discovery of this study. Shortcomings and limitations on the result interpretations and future direction of the research are reported in Sections 4 and 5, respectively.

**Table 5:** Pairwise Kruskal–Wallis *H*-test results for the acoustic biomarkers that were statistically significant (*p* < 0.05) in the TDU, RP, and SS tasks, respectively, within the six data sets analyzed.

**Statistics acoustic biomarkers**									
**Data set**	**Task**	**Biomarker**	**Sample** (***n***)		***H***	***p*-value**	**OB**	**η^2^**	**AUROC**
			**1**	**2**					
NLS	SS	SPTIME	CN (*n* = 33)	PD (*n* = 23)	8.65	0.01	↓	0.14	0.72
		SILDUR	CN (*n* = 33)	PD (*n* = 23)	7.87	0.02	↑	0.13	0.72
		SILVAR	CN (*n* = 33)	PD (*n* = 23)	7.68	0.02	↑	0.12	0.72
		F0STD	CN (*n* = 33)	PD (*n* = 23)	4.58	0.048	↓	0.07	0.67
		INTSTD	CN (*n* = 33)	PD (*n* = 23)	13.36	<0.001	↑	0.22	0.79
	RP	SILTIME	CN (*n* = 32)	PD (*n* = 22)	5.58	0.03	↑	0.09	0.69
		SILPERC	CN (*n* = 32)	PD (*n* = 22)	6.72	0.03	↑	0.11	0.69
		SILSPRAT	CN (*n* = 32)	PD (*n* = 22)	6.72	0.03	↑	0.11	0.71
		SILDUR	CN (*n* = 32)	PD (*n* = 22)	6.12	0.03	↑	0.10	0.71
		SILVAR	CN (*n* = 32)	PD (*n* = 22)	5.62	0.03	↑	0.09	0.70
		F0STD	CN (*n* = 32)	PD (*n* = 22)	5.35	0.03	↓	0.08	0.69
		INTSTD	CN (*n* = 32)	PD (*n* = 22)	5.17	0.03	↑	0.08	0.69
GermanPD	TDU	SILTIME	CN (*n* = 88)	PD (*n* = 88)	22.32	<0.001	↑	0.12	0.71
		SILPERC	CN (*n* = 88)	PD (*n* = 88)	32.27	<0.001	↑	0.17	0.75
		SILSPRAT	CN (*n* = 88)	PD (*n* = 88)	32.27	<0.001	↑	0.17	0.67
		SILDUR	CN (*n* = 88)	PD (*n* = 88)	27.63	<0.001	↑	0.14	0.73
		F0STD	CN (*n* = 88)	PD (*n* = 88)	16.31	<0.001	↓	0.09	0.68
		F1STD	CN (*n* = 88)	PD (*n* = 88)	7.93	0.007	↓	0.04	0.62
	RP	F0STD	CN (*n* = 88)	PD (*n* = 88)	27.52	<0.001	↓	0.14	0.73
		INTSTD	CN (*n* = 88)	PD (*n* = 88)	18.15	<0.001	↓	0.09	0.69
	SS	SPTIME	CN (*n* = 88)	PD (*n* = 88)	24.90	<0.001	↓	0.14	0.72
		SILDUR	CN (*n* = 88)	PD (*n* = 88)	7.69	0.01	↑	0.05	0.62
		F0STD	CN (*n* = 88)	PD (*n* = 88)	21.61	<0.001	↓	0.12	0.70
		INTSTD	CN (*n* = 88)	PD (*n* = 88)	33.63	<0.001	↑	0.20	0.75
Neurovoz	TDU	SPTIME	CN (*n* = 46)	PD (*n* = 43)	15.17	<0.001	↓	0.16	0.74
		SILPERC	CN (*n* = 46)	PD (*n* = 43)	11.60	0.001	↑	0.12	0.71
		SILSPRAT	CN (*n* = 46)	PD (*n* = 43)	11.60	0.001	↑	0.12	0.71
		SILVAR	CN (*n* = 46)	PD (*n* = 43)	7.03	0.01	↑	0.07	0.62
ItalianPVS	TDU	SPTIME	CN (*n* = 35)	PD (*n* = 28)	13.23	<0.001	↑	0.20	0.77
		SILTIME	CN (*n* = 35)	PD (*n* = 28)	14.15	<0.001	↑	0.22	0.78
		SILPERC	CN (*n* = 35)	PD (*n* = 28)	7.72	0.006	↑	0.11	0.71
		SILSPRAT	CN (*n* = 35)	PD (*n* = 28)	7.72	0.006	↑	0.11	0.71
		SILDUR	CN (*n* = 35)	PD (*n* = 28)	22.11	<0.001	↑	0.34	0.85
		SILVAR	CN (*n* = 35)	PD (*n* = 28)	14.47	<0.001	↑	0.23	0.78
		F0STD	CN (*n* = 35)	PD (*n* = 28)	10.56	<0.001	↓	0.16	0.74
	RP	INTSTD	CN (*n* = 35)	PD (*n* = 28)	32.79	<0.001	↑	0.52	0.92
		SILTIME	CN (*n* = 21)	PD (*n* = 26)	7.79	0.02	↑	0.15	0.74
		SILPERC	CN (*n* = 21)	PD (*n* = 26)	6.81	0.02	↑	0.12	0.72
		SILSPRAT	CN (*n* = 21)	PD (*n* = 26)	6.81	0.02	↑	0.12	0.72
	RP	SILDUR	CN (*n* = 21)	PD (*n* = 26)	10.85	0.01	↑	0.22	0.78
		SILVAR	CN (*n* = 21)	PD (*n* = 26)	5.54	0.03	↑	0.11	0.70
		F0STD	CN (*n* = 21)	PD (*n* = 26)	6.37	0.01	↑	0.12	0.72
		INTSTD	CN (*n* = 21)	PD (*n* = 26)	17.23	<0.001	↑	0.36	0.86
		SILTIME	CN (*n* = 16)	PD (*n* = 20)	9.92	0.005	↑	0.12	0.82
CzechPD		SILPERC	CN (*n* = 16)	PD (*n* = 20)	9.75	0.002	↑	0.25	0.85
		SILSPRAT	CN (*n* = 16)	PD (*n* = 20)	12.25	0.002	↑	0.33	0.85
		SILDUR	CN (*n* = 16)	PD (*n* = 20)	8.21	0.009	↑	0.21	0.79
		F0STD	CN (*n* = 16)	PD (*n* = 20)	5.92	0.04	↓	0.14	0.74
		INTSTD	CN (*n* = 16)	PD (*n* = 20)	4.69	0.04	↓	0.10	0.72
		SILVAR	CN (*n* = 16)	PD (*n* = 20)	4.69	0.04	↓	0.10	0.72
	SS	SILTIME	CN (*n* = 16)	PD (*n* = 20)	14.44	0.001	↑	0.39	0.88
		SILPERC	CN (*n* = 16)	PD (*n* = 20)	11.33	0.002	↑	0.30	0.84
		SILSPRAT	CN (*n* = 16)	PD (*n* = 20)	11.33	0.002	↑	0.30	0.84
		SILDUR	CN (*n* = 16)	PD (*n* = 20)	11.33	0.002	↑	0.30	0.84
		F0STD	CN (*n* = 16)	PD (*n* = 20)	6.41	0.03	↓	0.16	0.75
GITA	RP	F0STD	CN (*n* = 50)	PD (*n* = 50)	8.46	0.01	↓	0.08	0.67
	TDU	F0STD	CN (*n* = 50)	PD (*n* = 50)	7.46	0.009	↓	0.06	0.57
		INTSTD	CN (*n* = 50)	PD (*n* = 50)	7.90	0.009	↓	0.07	0.57
		F1STD	CN (*n* = 50)	PD (*n* = 50)	5.18	0.02	↓	0.04	0.56

OB, observed behavior.

↑ means increasing, ↓ means decreasing.

**Table 6:** Pairwise Kruskal–Wallis *H*-test results for the linguistic and cognitive biomarkers that were statistically significant (*p* < 0.05) in the TDU, RP, and SS tasks, respectively, within the six data sets analyzed.

**Statistics linguistic and cognitive biomarkers**									
**Data set**	**Task**	**Biomarker**	**Sample** (***n***)		***H***	***p*-value**	**OB**	**η^2^**	**AUROC**
			**1**	**2**					
NLS	SS	RHYSTD	CN (*n* = 33)	PD (*n* = 23)	5.37	0.02	↓	0.08	0.67
	RP	RHYSTD	CN (*n* = 32)	PD (*n* = 22)	3.88	0.048	↑	0.06	0.65
GermanPD	SS	WORDCNT	CN (*n* = 88)	PD (*n* = 88)	16.22	<0.001	↓	0.08	0.68
		SENTCNT	CN (*n* = 88)	PD (*n* = 88)	7.86	0.005	↓	0.04	0.62
		NOUNCNT	CN (*n* = 88)	PD (*n* = 88)	14.11	<0.001	↓	0.07	0.66
		AUXCNT	CN (*n* = 88)	PD (*n* = 88)	11.85	0.001	↓	0.06	0.65
		NPCNT	CN (*n* = 88)	PD (*n* = 88)	9.96	0.003	↓	0.05	0.64
		PPCNT	CN (*n* = 88)	PD (*n* = 88)	13.50	<0.001	↓	0.07	0.64
		RHYSTD	CN (*n* = 88)	PD (*n* = 88)	19.35	<0.001	↓	0.10	0.69
Neurovoz	TDU	RHYSTD	CN (*n* = 46)	PD (*n* = 43)	15.27	<0.001	↓	0.16	0.74
	SS	WORDCNT	CN (*n* = 46)	PD (*n* = 43)	10.56	0.01	↓	0.11	0.74
		SENTCNT	CN (*n* = 46)	PD (*n* = 43)	13.37	0.003	↓	0.14	0.83
		NOUNCNT	CN (*n* = 46)	PD (*n* = 43)	7.06	0.01	↓	0.07	0.74
		VERBCNT	CN (*n* = 46)	PD (*n* = 43)	11.22	0.006	↓	0.11	0.80
		AUXCNT	CN (*n* = 46)	PD (*n* = 43)	7.11	0.01	↓	0.07	0.74
		NPCNT	CN (*n* = 46)	PD (*n* = 43)	8.84	0.01	↓	0.09	0.77
		VPCNT	CN (*n* = 46)	PD (*n* = 43)	6.91	0.01	↓	0.07	0.74
		RHYSTD	CN (*n* = 46)	PD (*n* = 43)	6.01	0.01	↓	0.06	0.72
		IU	CN (*n* = 46)	PD (*n* = 43)	5.21	0.02	↓	0.05	0.70
ItalianPVS GITA	RP	RHYSTD	CN (*n* = 21)	PD (*n* = 26)	16.99	<0.001	↑	0.35	0.81
	TDU	RHYSTD	CN (*n* = 21)	PD (*n* = 26)	3.91	0.047	↑	0.06	0.67
	TDU	RHYSTD	CN (*n* = 50)	PD (*n* = 50)	4.07	0.04	↓	0.03	0.62

OB, observed behavior.

↑ means increasing, ↓ means decreasing.

**Table 7:** Results of Spearman's rank correlation (ρ) with corresponding *p*-values after the FDR correction.

**Data set**	**Task**	**Biomarker**	**UPDRS-III**		**UPDRS-III.I (speech)**		**H&Y**	
						***p*-value**	**ρ**	***p*-value**	**ρ**	***p*-value**	**ρ**
NLS	RP	SILTIME	0.01	0.49 (↑)	–	–	–	–
		SILPERC	0.01	0.51 (↑)	–	–	–	–
		SILSPRAT	0.01	0.51 (↑)	–	–	–	–
GermanPD	RP	SPTIME	–	–	–	–	0.008	0.33 (↑)
		SILTIME	–	–	–	–	0.007	0.35 (↑)
		SILSPRAT	–	–	–	–	0.007	0.31 (↑)
		SILDUR	–	–	–	–	0.007	0.32 (↑)
	TDU	RHYSTD	–	–	–	–	<0.001	0.38 (↑)
CzechPD	RP	INTSTD	0.02	−0.60 (↓)	–	–	–	–
Neurovoz	TDU	F0STD	0.04	−0.43 (↓)	–	–	–	–
		F1STD	0.04	0.42 (↑)	–	–	–	–
	SS	SILDUR	0.02	0.67 (↑)	–	–	–	–
ItaliaPVS	TDU	SPTIME	–	–	0.02	−0.47 (↓)	–	–
		SILTIME	–	–	0.003	0.63 (↑)	–	–
		SILPERC	–	–	0.003	0.63 (↑)	–	–
		SILSPRAT	–	–	0.003	0.63 (↑)	–	–
		SILVAR	–	–	0.048	0.40 (↑)	–	–
		RHYSTD	–	–	0.018	0.48 (↑)	–	–
	RP	RHYSTD	–	–	<0.001	0.67 (↑)	–	–

Results are reported only for those biomarkers whose *p*-values were significant. In performing the correlation analysis on ItalianPVS, we used only the UPDRS-III scores related to the speech assessment (UPDRS-III.I) since the scores for the other test sections were unavailable.

↑ means increasing, ↓ means decreasing.

### 3.1. Acoustic biomarkers

As we have noted early, neurodegeneration in PD has dopaminergic and non-dopaminergic contributions that most immediately impacts the striatum but also have downstream cortical effects. Consequently, it is uncertain whether a given biomarker's performance may be solely due to altered dopamine transmission. One possible reductionistic mechanism may be the loss of dopaminergic neurons in the substantia nigra results in generalized muscle rigidity, which subsequently affects laryngeal muscular tone (phonatory subsystem). This fact may increase the laryngeal tension (physiological correlate), resulting in decreased F0 speech variability (23, 61, 62). Previous studies have shown that F0 variability tends to be significantly lower for subjects with PD than CN subjects and can significantly increase after patients receive dopaminergic medications (35, 63). In contrast, Cavallieri et al. (64) found a greater impact of L-Dopa *via* levodopa-induced dyskinesia on speech performance rather than during L-Dopa untreated states at least in persons with advanced disease, which is not well-represented in our data sets. Subjects with PD also exhibit speech rate abnormalities characterized by excessive and longer speech pauses (29, 65–67). The results reported on monoloudness are controversial: some studies have reported a reduced amplitude variability in patients with PD (68), while others have not identified any significant difference between PD and CN groups (67, 69). Phonatory and articulatory deficits have also been extensively studied. Concerning phonation, jitter, shimmer, and Harmonic to Noise Ratio (HNR) represent typical measures. Previous studies have employed a sustained phonation of vowels to extract these biomarkers and obtained significant differences between PD and CN groups for the three measurements, where jitter and shimmer were higher and HNR was lower in patients with PD vs. CN participants (70–72). Concerning articulation, formant centralization ratio (FCR), the duration of voiced segments (DVS), voice onset time (VOT), and VSA have been analyzed, among others. Results showed that FCR, DVS, and VOT tended to be greater in participants with PD than in CN participants (73–75), while reduced values of VSA in articulation were reported for participants with PD vs. CN participants (28, 36). In some cases, the inter-subject variability in phonation, articulation, and prosody might act as a confounding variable, as shown by Rusz et al. (76). In that study, combining these biomarkers allowed a good discriminability of the newly diagnosed PD group (confirmed by dopamine transporter imaging) from the CN group. Moreover, a significant gender difference was reported, with female patients having greater vocal control than men patients. The robustness of F0, pause-related biomarkers, and jitter was confirmed in earlier multilingual studies (29, 30). In our study, articulatory and phonatory speech aspects were not considered.

**Figure 1:**
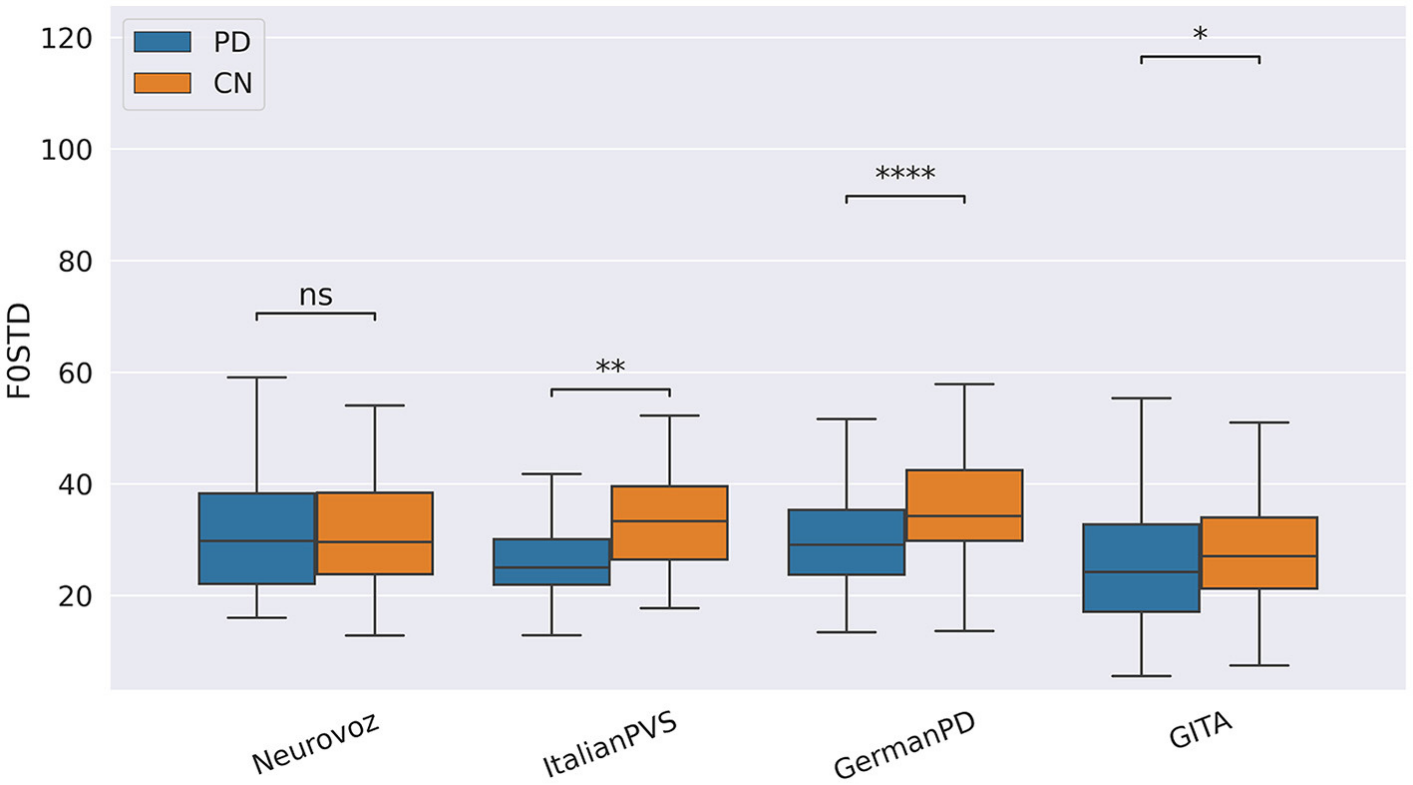
Boxplots with statistical annotations representing the standard deviation of F0 (F0STD) on Neurovoz, ItalianPVS, GermanPD, and GITA from the TDU task. Horizontal lines represent the median, the top ends represent the upper quartile, and the bottom ends represent the lower quartile. Statistically significant differences between groups after Benjamini–Hochberg correction for F0STD are reported in Table 5 and highlighted below using asterisks. F0STD, F0 standard deviation; CN, controls; PD, Parkinson's disease; TDU, text dependent utterance; ns, not significant.

As suggested by the results reported in Table 5, participants with PD exhibited monopitch (i.e., significantly lower F0STD) during the RP task on NLS, GermanPD, ItalianPVS, CzechPD, and GITA. Similar results were observed in the analysis of the spoken responses to the SS task on NLS and CzechPD and the TDU task on GermanPD, ItalianPVS, and GITA (see Figure 1). Aligned with these results, F0STD showed a negative correlation with the UPDRS-III scores in Neurovoz. As such, this biomarker met the requirements for robustness and confirmed results reported in previous monolingual and multilingual studies, extending them to other languages (i.e., German and Castilian Spanish). Even though participants with PD and CN participants in NLS, GermanPD, ItalianPVS, and CzechPD were not perfectly matched in terms of gender, the gender variable should not have influenced this result as we measured the standard deviation of F0 and not its mean, which is sensitive to the gender distributions of the experimental groups.

During the RP tasks, speech loudness variability (INTSTD) was significant on NLS, GermanPD, ItalianPVS, and CzechPD. However, Italian and American participants with PD reported higher INTSTD compared to their respective CN groups. Consequently, INTSTD did not meet the robustness conditions since its observed behavior differed from its expected cross-lingual behavior. A similar observation applies to the standard deviation of the first formant (F1STD). As expected, this biomarker was significantly lower for the PD group than for the CN group on GITA and GermanPD during the TDU task. However, F1STD showed a significant positive correlation on Neurovoz in the TDU task. As such, this biomarker did not meet the robustness conditions. Even though we applied loudness normalization before computing intensity-related biomarkers, the experimental results might have been partially influenced by the adoption of different recording conditions (e.g., headset microphone, where the mouth-to-diaphragm distance is constant vs. microphone on a tripod where this distance could be variable within a single recording). Even in the multi-lingual analysis performed by Kovac et al. (30), INTSTD and F1STD did not meet the robustness criteria.

During the RP and TDU tasks, speech time (SPTIME) was significantly higher in almost all the data sets analyzed (as expected), except in Neurovoz, where participants with PD showed a significantly lower SPTIME than CN participants. In this respect, we observed that while reading, subjects with PD do not just insert (part of) words to repeat mispronounced items or to correct themselves, but they also skip some words due to reading difficulties. In Neurovoz, this phenomenon can be motivated by the fact that participants were not required to read the short sentences from a printed sheet. However, they listened to them from a recording of a standard speaker and repeated them out loud. In this process, they might have forgotten some words to utter, which could have caused a significantly lower SPTIME. Thus, this result does not confirm previous findings (46) that showed that participants with PD, when reading, have a higher SPTIME due to word repetitions and self-corrections. However, two different acquisition protocols are probably the cause here; thus, this biomarker does not exhibit task robustness rather than language robustness.

**Figure 2:**
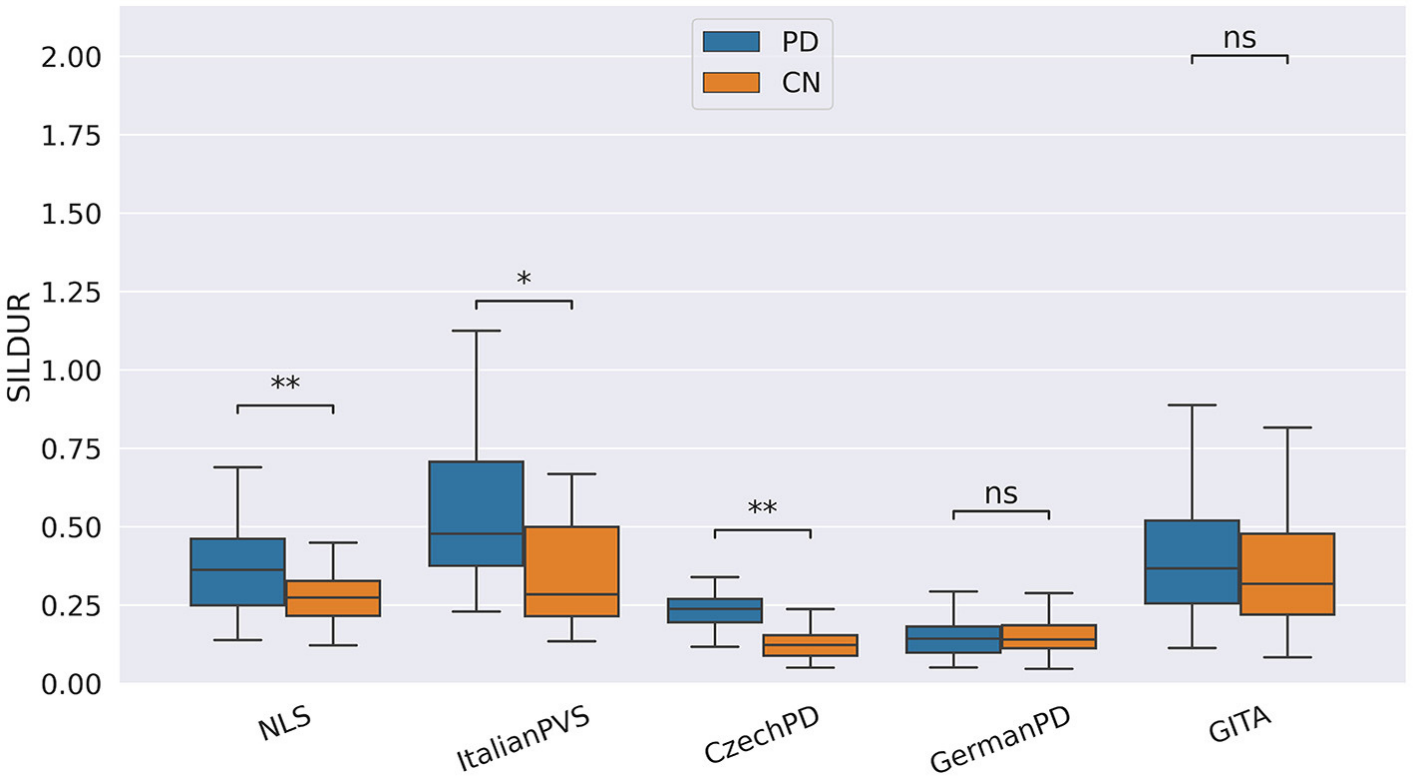
Boxplots with statistical annotations representing the silence duration (SILDUR) on NLS, Neurovoz, CzechPD, GermanPD, and GITA from the RP task. Horizontal lines represent the median, the top ends represent the upper quartile, and the bottom ends represent the lower quartile. Statistically significant differences between groups after Benjamini–Hochberg correction for SILDUR are reported in Table 5 and highlighted below using asterisks. SILDUR, silence duration; CN, controls; PD, Parkinson's disease; SILDUR, silence duration; SS, spontaneous speech; ns, not significant.

Pause-related biomarkers such as the amount of silent time (SILTIM), the silent time percentage (SILPERC), the silence-to-speech ratio (SILSPRAT), and the silence time variability (SILVAR) were significantly higher during the TDU and RP tasks on NLS, GermanPD, Neurovoz, ItalianPVS, and CzechPD due to the difficulties imposed by the two reading tasks. Even though these four biomarkers were not significant on GITA, their behaviors were consistent with those observed in the other data sets and aligned with the expected behavior. Similarly, the length of the pauses (SILDUR) was significantly higher on NLS, GermanPD, ItalianPVS, and CzechPD during the SS, TDU, and RP tasks, respectively (see Figure 2). SILTIM, SILPERC, SILSPRAT, SILVAR, and SILDUR also behaved as expected in the correlation analysis, where they exhibited a positive correlation with both UPDRS-III, UPDRS-III.I, and H&Y scores on NLS, Neurovoz, GermanPD, and ItalianPVS, respectively. No significant difference between the CN group and the PD group was observed on GITA with respect to the silence-time and speech-time-related biomarkers. This result can be motivated by the shorter length of the texts adopted in the TDU and RP tasks within the GITA data set, which prevented observing a significant number of hesitations and pauses as in the other data sets. In general, the cognitive load in some tasks can be greater than in others. For instance, asking a participant to describe what she did during the morning could be easier than providing an exhaustive and coherent picture description within a time limit of 60 *s*. Moreover, the total number of TDUs in GITA is six, while in other data sets, such as ItalianPVS is ten. Therefore, if a data set has relatively complex tasks, it might be more than linearly expected to lead to greater values in the pause-related features. Overall, these findings are aligned with previous studies that reported more frequent and prolonged pauses in the spontaneous production and read speech of PD individuals across different languages (29).

### 3.2. Linguistic and cognitive biomarkers

Studies focusing on language-related impairments reported that persons with PD demonstrated impairments in syntax and semantics, especially in producing and processing action words compared with non-action words (47). While a relationship between action-verb or semantic processing and bradykinesia may be serendipitous, an underlying informatic dysfunction in *action* conceptually (as *qualia*) would be intriguing. A relationship between action words and physical movements may explain why individuals with PD have more difficulty producing and processing action words than non-action words. In this respect, the explanation proposed by the *Embodied Cognition* (EC) theory (77, 78) is that the categories of content are represented in different brain regions depending on the sensory and motor processes involved in the acquisition of the content (79). Thus, the action word deficit in PD may be related to impaired input to the striatum from the motor and associated cortex. Some studies have found that individuals with PD exhibit difficulties in both actions and object naming (80) and improved action naming more than object naming upon the stimulation of the subthalamic nucleus- a common target of deep brain stimulation to improve the motor function (81).

Moreover, previous studies suggest that subjects with PD and related disorders exhibit reduced information content in their descriptions when required to mention the main characters/events displayed in a picture (31, 82–84). These results are consistent with findings reporting that subjects with PD have selective deficits in naming and processing action verbs and object nouns since the lower level of informativeness can be originated, at least partially, by the impairments in specific syntactic categories (e.g., nouns and verbs). Individuals with PD also demonstrated difficulty modulating speech rhythm or timing organization (51, 85), a phenomenon that contributes to a more static rhythm in spontaneous production and that makes the semantics of the discourse less clear. Altogether, these findings have never been validated before within a multi-lingual study.

**Figure 3:**
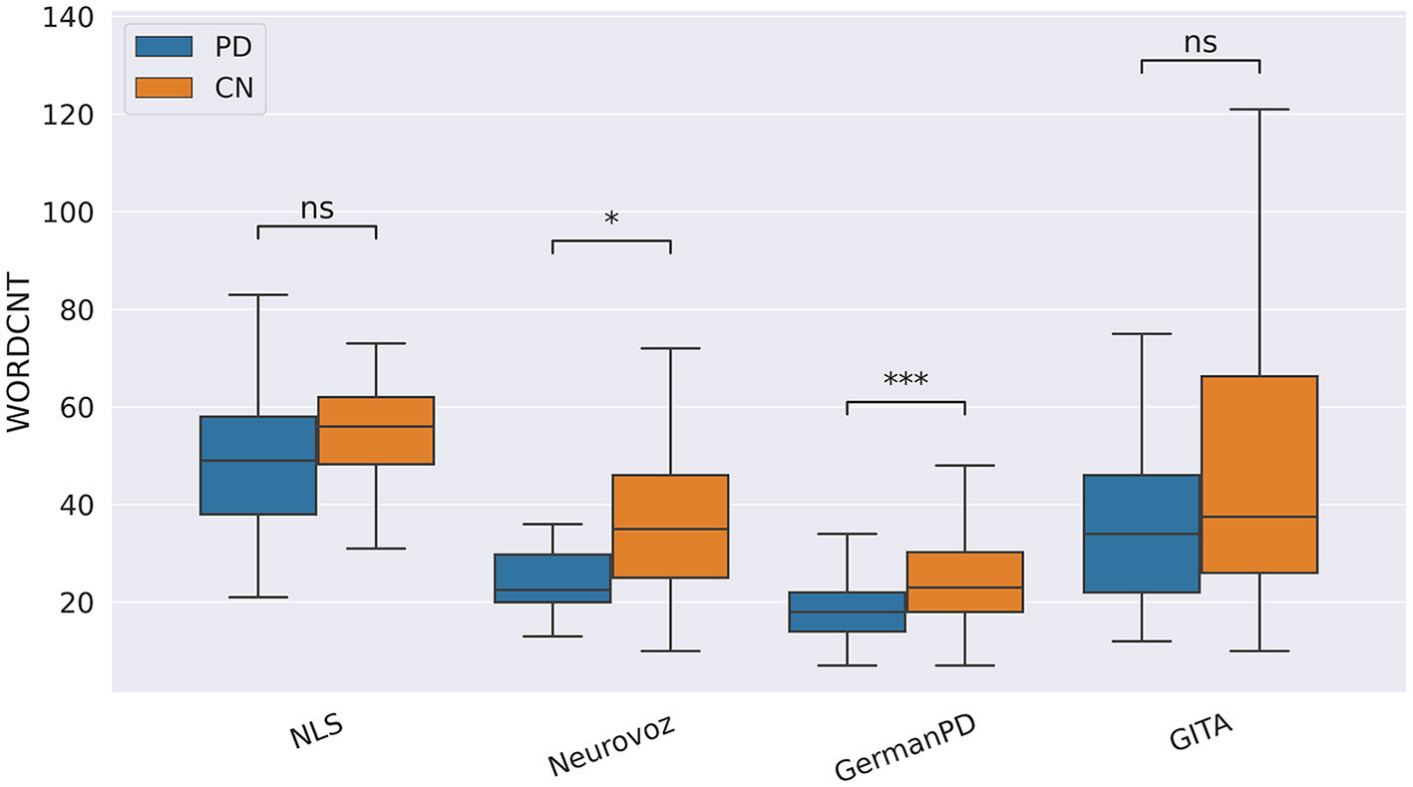
Boxplots with statistical annotations representing the word count (WORDCNT) on NLS, Neurovoz, GermanPD, and GITA from the SS task. Horizontal lines represent the median, the top ends represent the upper quartile, and the bottom ends represent the lower quartile. Statistically significant differences between groups after Benjamini–Hochberg correction for WORDCNT are reported in Table 6 and highlighted below using asterisks. WORDCNT, word count; CN, controls; PD, Parkinson's disease; SS, spontaneous speech; ns, not significant.

**Figure 4:**
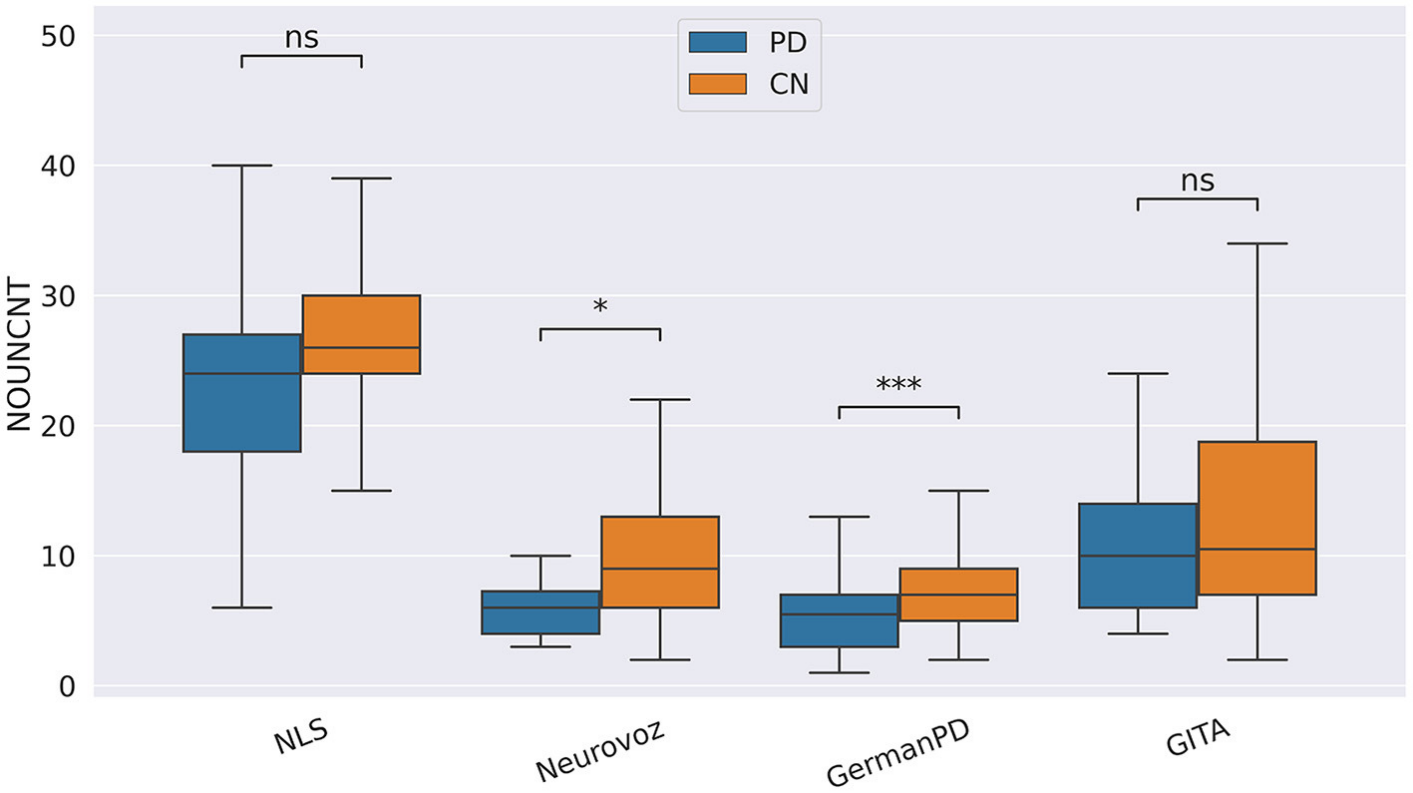
Boxplots with statistical annotations representing the noun count (NOUNCNT) on NLS, Neurovoz, GermanPD, and GITA from the SS task. Horizontal lines represent the median, the top ends represent the upper quartile, and the bottom ends represent the lower quartile. Statistically significant differences between groups after Benjamini–Hochberg correction for NOUNCNT are reported in Table 6 and highlighted below using asterisks. NOUNCNT, noun count; CN, controls; PD, Parkinson's disease; SS, spontaneous speech; ns, not significant.

**Figure 5:**
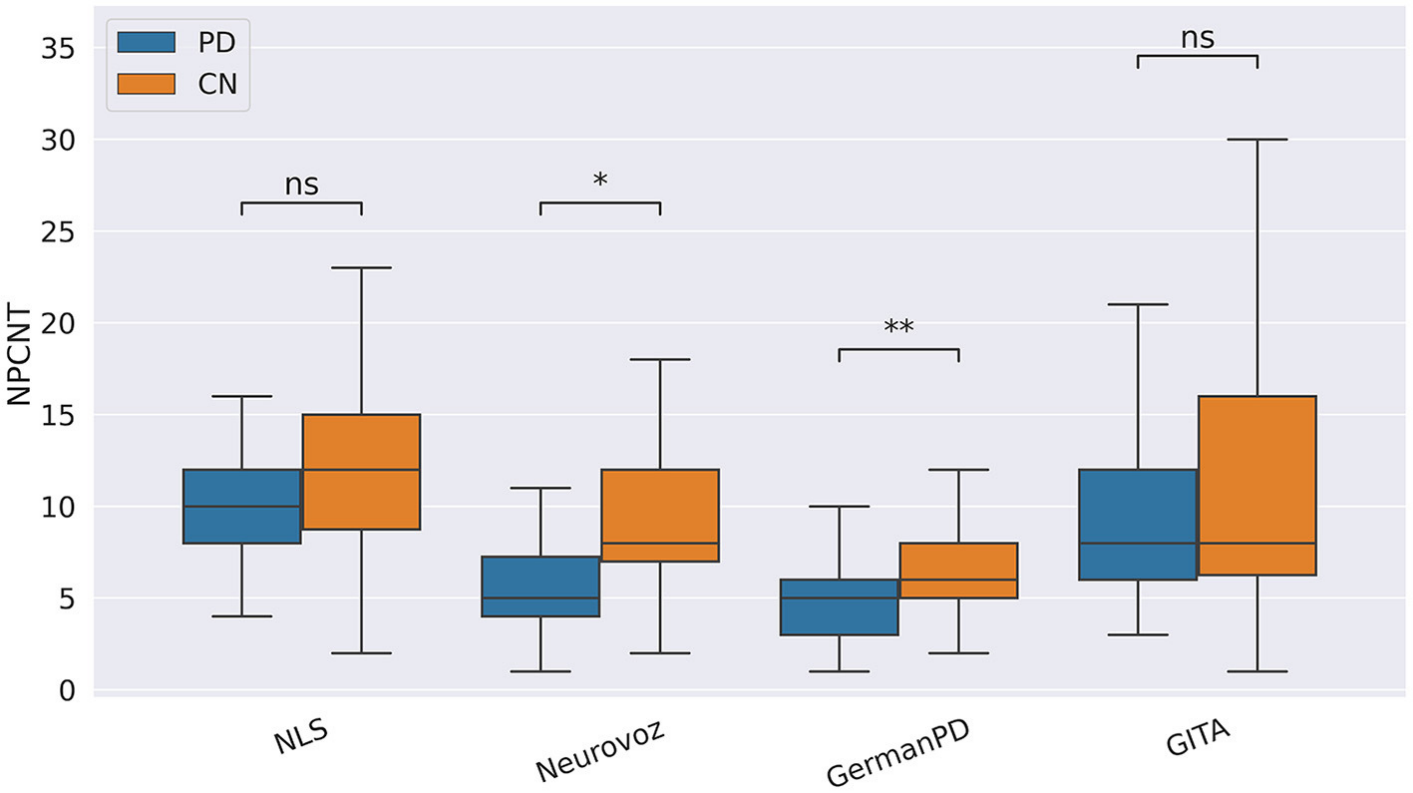
Boxplots with statistical annotations representing the noun phrase count (NPCNT) on NLS, Neurovoz, GermanPD, and GITA from the SS task. Horizontal lines represent the median, the top ends represent the upper quartile, and the bottom ends represent the lower quartile. Statistically significant differences between groups after Benjamini–Hochberg correction for NPCNT are reported in Table 6 and highlighted below using asterisks. NPCNT, noun phrase count; CN, controls; PD, Parkinson's disease; SS, spontaneous speech; ns, not significant.

**Figure 6:**
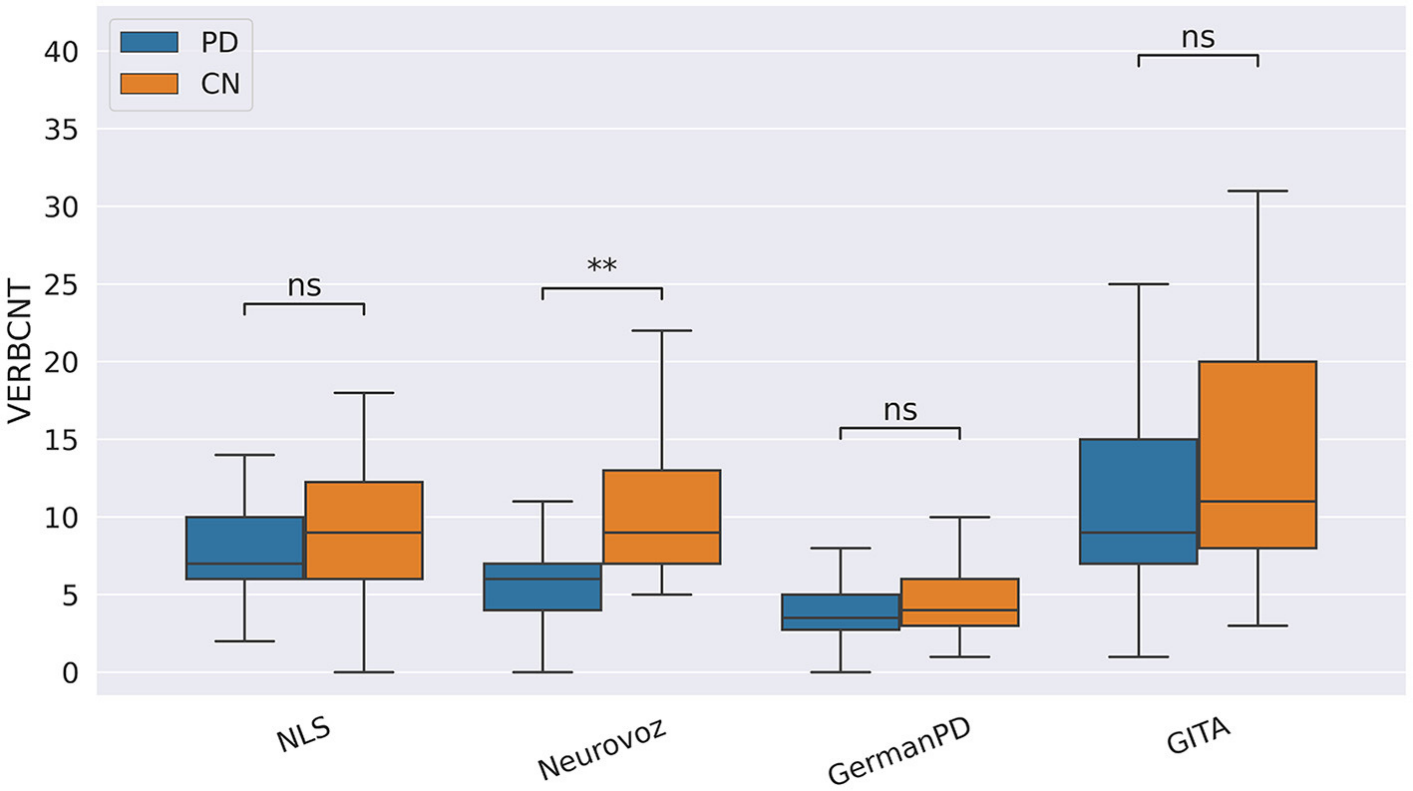
Boxplots with statistical annotations representing the verb count (VERBCNT) on NLS, Neurovoz, GermanPD, and GITA from the SS task. Horizontal lines represent the median, the top ends represent the upper quartile, and the bottom ends represent the lower quartile. Statistically significant differences between groups after Benjamini–Hochberg correction for VERBCNT are reported in Table 6 and highlighted below using asterisks. VERBCNT, verb count; CN, control group; PD, Parkinson's disease; SS, spontaneous speech; ns, not significant.

**Figure 7:**
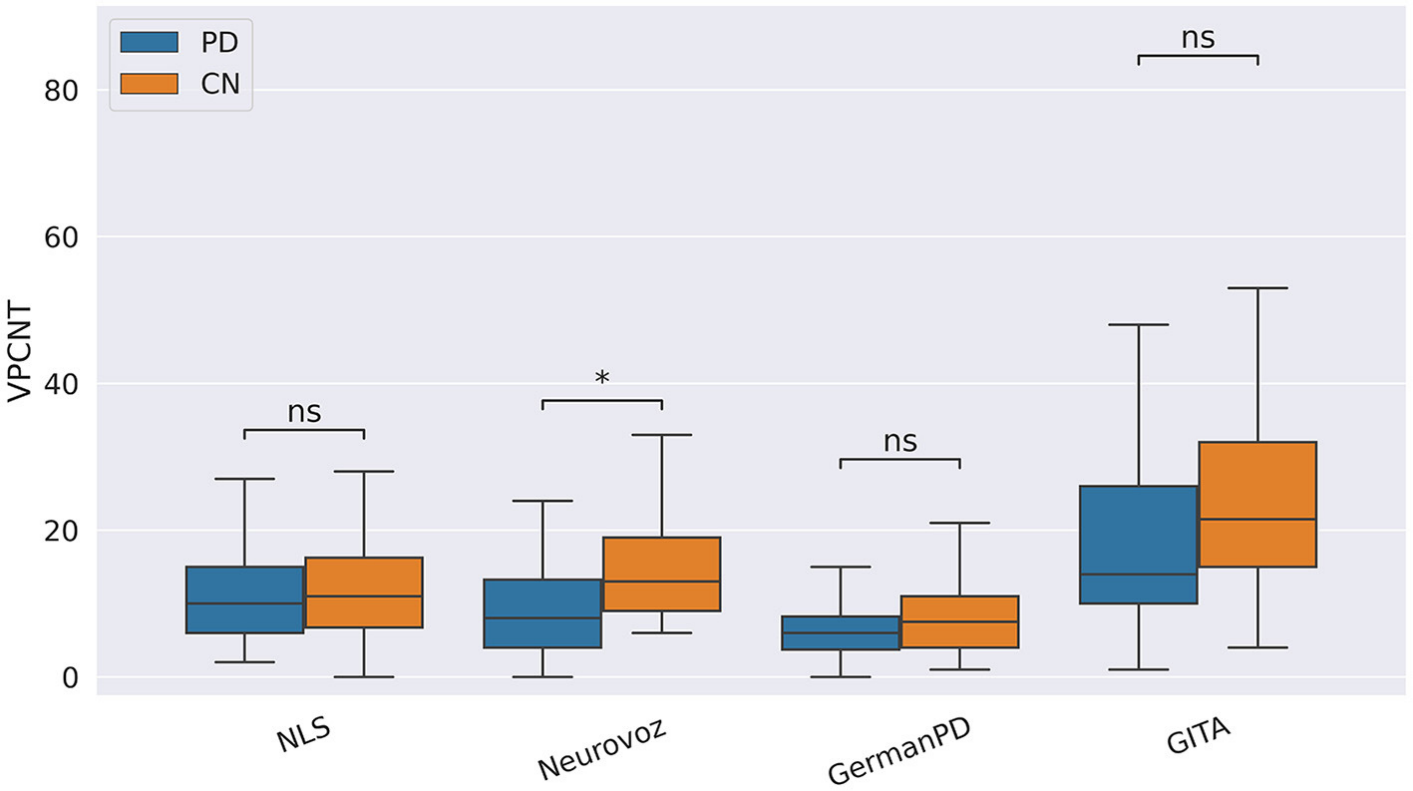
Boxplots with statistical annotations representing the verb phrase count (VPCNT) on NLS, Neurovoz, GermanPD, and GITA from the SS task. Horizontal lines represent the median, the top ends represent the upper quartile, and the bottom ends represent the lower quartile. Statistically significant differences between groups after Benjamini–Hochberg correction for VPCNT are reported in Table 6 and highlighted below using asterisks. VPCNT, verb phrase count; CN, control group; PD, Parkinson's disease; SS, spontaneous speech; ns, not significant.

In our study, participants with PD tended to deliver shorter narratives during the SS task. This phenomenon was reflected by a significantly lower amount of speech time (SPTIME) observed on NLS, GermanPD, and CzechPD. Aligned with this result, the number of word tokens (WORDCNT; see Figure 3) and sentences (SENTCNT) were significantly lower for participants with PD than for CN participants on GermanPD and Neurovoz data sets. In the other data sets, even though these two biomarkers were not significant, their behaviors were those expected. Moreover, subjects with PD tended to use a lower number of nouns (NOUNCNT), auxiliaries (AUXCNT), and noun phrases (NPs) on GermanPD and Neurovoz. On Neurovoz, the number of verbs (VERBCNT) and verb phrases (VPCNT) were significantly lower for PD vs. CN participants. Figures 4–7 report the distributions of NOUNCNT, NPCNT, VERBCNT, and VPCNT, respectively, from the SS task. Even though VERBCNT and VPCNT were significant and behaved as expected, since they were significant only on Neurovoz, we did not consider them robust. On the one hand, the picture description task contained in Neurovoz requires participants to describe what the four main characters in the picture are doing. As such, this task is implicitly designed to assess any deficit in object and action naming. On the other hand, in the other data sets (except NLS), during the SS task, participants are required to provide a general description of their daily routine or to deliver a generic narrative. Thus, these tasks do not directly assess any deficit in action naming. This finding suggests the possibility already introduced in previous studies: subjects with PD may develop a deficit in noun processing and object naming (80). In addition, this finding extends results obtained in monolingual studies to a multi-lingual cohort, showing their robustness. Not having found similar results on CzechPD could have been caused by the fact that the linguistic analysis could not be performed on CzechPD because no pre-trained models were available for the Czech language. We expect that biomarkers like SENTCNT and WORDCNT could have been significant also in CzechPD since the acoustic analysis confirmed that participants with PD delivered shorter narratives than CNs.

**Figure 8:**
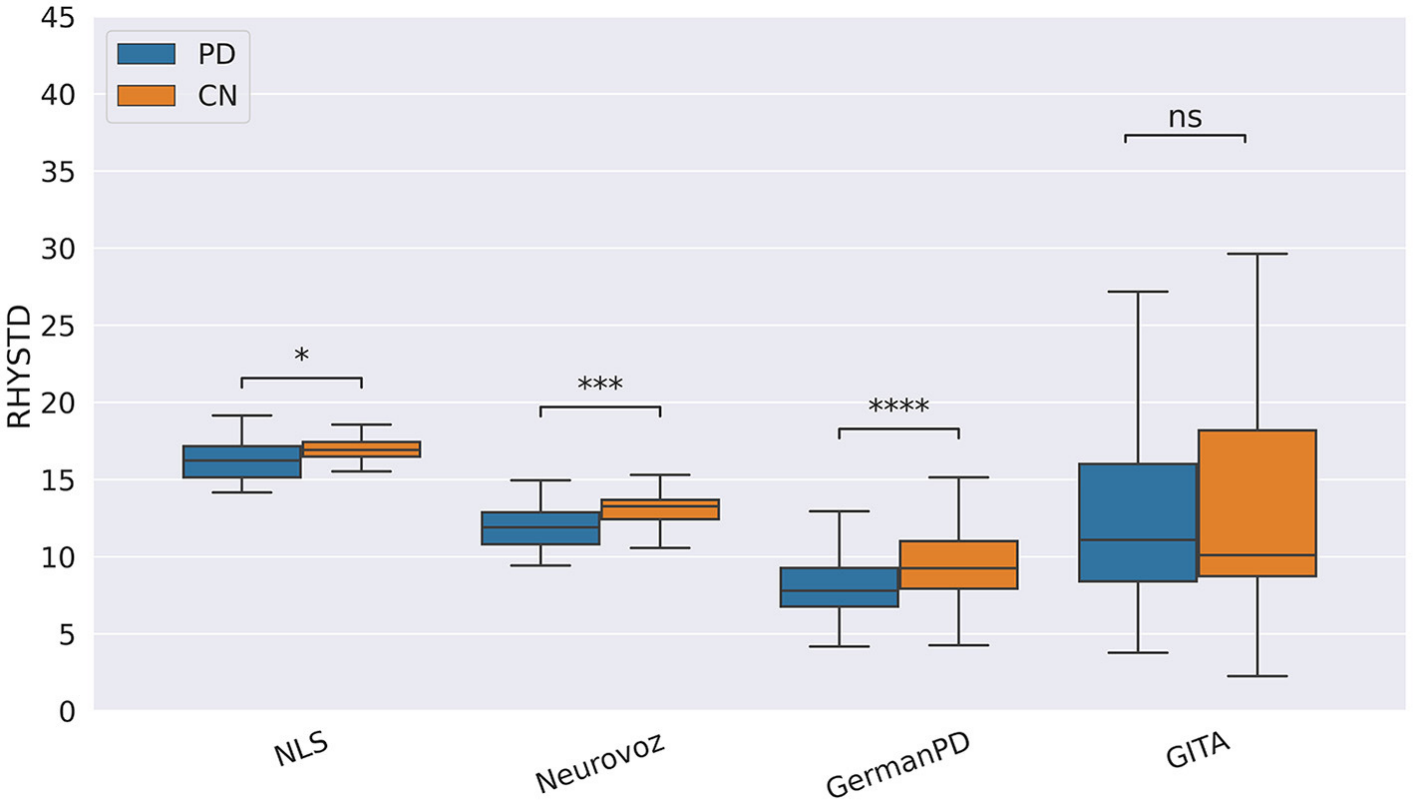
Boxplots representing the rhythm standard deviation (RHYSTD) on NLS, Neurovoz, GermanPD, and GITA from the SS task. Horizontal lines represent the medians, the top ends represent the upper quartiles, and the bottom ends represent the lower quartiles. Statistically significant differences between groups after Benjamini–Hochberg correction for RHYSTD are reported in Table 6 and highlighted below using asterisks. RHYSTD, rhythm standard deviation; CN, control group; PD, Parkinson's disease; SS, spontaneous speech; ns, not significant.

Concerning the cognitive biomarkers, subjects with PD tended to deliver less informative narratives. During the picture description task, the number of correct informational units (IU) was significantly lower for subjects with PD on Neurovoz but not on NLS. Thus, we did not consider IU a robust biomarker. To conclude, during the TDU and RP tasks, the standard deviation of the speech rhythm (RHYTSTD) was significantly higher for the PD group than for the CN group on ItalianPVS and NLS, respectively (as predicted), but significantly lower for the PD group than for the CN group on Neurovoz and GITA. As such, the behavior of this biomarker differed from its expected cross-lingual behavior. However, the analysis of two different types of tasks (short sentences vs. read passages) is probably the cause here; thus, this biomarker might not show the task robustness rather than the language robustness. On the other hand, during the SS task, RHYSTD was significantly lower for the PD group vs. the CN group on NLS, Neurovoz, and GermanPD (see Figure 8). Thus, RHYSTD exhibited robustness on the SS task. In addition to being aligned with our predictions, this result extends to a multi-lingual cohort previous findings by showing that subjects with PD exhibit more static rhythm in everyday conversations.

## 4. Other factors and possible limitations

We have identified some limitations and factors that can influence the results and conclusions of this and other similar studies. These are listed in this section. First, the data sets analyzed vary in size, gender distribution, and case severity, so the classes are not optimally balanced. Self-selection and survival biases are common in PD research, whereas younger, healthier, and more motivated subjects are easier to recruit and monitor longitudinally, while advanced, non-ambulatory subjects inevitably drop out (86). This is a recognized limitation, and we agree with Vaswani et al. (86) that technological innovation may improve diversity in subject recruitment.

In all the data sets (except in CzechPD), participants received dopaminergic medication right before starting the recording session (i.e., 1–5 h early), which could have improved their speech and language performances. However, in the end, this factor did not seem to have exerted a particular influence on the experimental results since CzechPD did not turn out to be the data set with the higher number of significant biomarkers. A higher number of robust biomarkers were reported on GermanPD and Neurovoz. This result could be explained by the fact that even though the different cohorts of participants with PD were distributed in the same range of disease stage (i.e., H&Y range 1–3), participants in GermanPD and Neurovoz were diagnosed with PD earlier than those recorded in NLS and CzechPD. As such, they could have more visible manifestations of the disorder in language and speech. The fact that Czech participants with PD were at earlier stages of the disorder is also reflected by the fact that they reported a significantly lower average UPDRS score than all the PD groups in the other data sets.

As speech-based interpretable biomarkers may help clinicians diagnose PD and monitor the disorder's evolution, it might have been better to focus on different PD subpopulations (76), namely, prodromal PD, early PD (e.g., H&Y scores 1–2), and moderate-advanced PD (H&Y score equal or greater than 3). Identifying speech disorder sub-types may improve the understanding of mechanisms underlying dysarthria in PD and benefit decisions regarding speech therapies to follow. In this study, grouping participants with PD in different sub-types could have been difficult because more UPDRS and diagnosis detailed information was missing for most data sets. For instance, the H&Y scale was missing for ItalianPVS. Second, with respect to the other five data sets in which the H&Y scale was available, we observed that the different cohorts reported a similar H&Y range (1–3) even though the time since diagnosis did vary considerably from one data set to another. As such, a subdivision solely based on H&Y would have probably not been representative since most of the subjects should have been grouped in the early-moderate subgroup.

Another limitation might be represented by the different acquisition protocols and hardware systems adopted in the data collection phase. For instance, while in NLS, Neurovoz, and GermanPD, speech samples were recorded using a headset, in GITA, CzechPD, and ItalianPVS, and speech samples were recorded with a microphone separated from the participants (i.e., mounted on a tripod). Using a microphone on a tripod allows fluctuations in the distance between the speaker's mouth and the microphone. This fact could have affected the computation of intensity variability (even though normalization was applied). As previously observed by Rusz et al. (87), good practice would require using a head-mounted microphone so that the mouth-to-microphone distance can be reduced and maintained constant at 4 cm. This way, the speech should be at least 10 dB greater than background noise. If a standard microphone placement 30 cm away from the mouth is used, the speech intensity level is ~35 dB, and the background noise and reverberation could be high, except in acoustically controlled conditions.

In addition, although we analyzed biomarkers extracted from the same task typology, comparing the results across data sets can still be problematic, given the differences between variants of the same task. In general, as observed in Section 3, if a database has relatively more complex tasks than others in cognitive load or task length, this can impact the results and the comparison across languages. Moreover, not the same clinical data (e.g., UPDRS III, UPDRS III.I, and H&Y scale) are available for each data set considered. This missing information could have prevented observing a significant correlation between the biomarkers and the clinical scores and influenced comparing the results obtained on different data sets.

Concerning the NLS data set collected by the authors of this study, some further observations should be reported. First, the participants recorded in NLS have been diagnosed with PD more recently than in GITA and GermanPD. Second, all the participants were required to wear surgical masks during the recording session to meet the COVID-19 safety requirements. Third, in the SS task, we imposed a time limit of 60 s for the participants to complete the task. This time limit is not imposed on the SS task collected in the other studies, meaning that the spontaneous speech samples could be longer and result in the extraction of more significant biomarkers. These aspects can motivate the lower number of significant biomarkers discovered on NLS. However, it is worth noting that even though a surgical mask could have modified the channel, it did that equally for all participants. Moreover, our study employed mask models that minimally interfered with jaw and lip movement. Moreover, the results of the linguistic analysis could not have been influenced by the presence of the mask since the syntactic and semantic abilities of the participants do not change depending on whether an individual wears a mask or not.

## 5. Conclusions and future work

In this study, we analyzed the behavior of a composite array of interpretable biomarkers encoding acoustic, linguistic, and cognitive information. The aim was to explore the effectiveness of these biomarkers in modeling characteristic patterns occurring in PD and assess their discriminatory power and language independence. Our statistical analysis showed that 13 of our biomarkers met the robustness conditions. In particular, the spontaneous production of participants with PD was characterized by shorter narratives in terms of speech time, number of words, nouns, auxiliaries, and NPs. Longer and more frequent pauses and a lower F0 variability (monotonic pitch) were observed in both spontaneous and read speech. A more static speech rhythm characterized spontaneous production only. Hence, this study provides insights into the objective assessment of PD from language and speech. It also represents one of the first studies that leverages the concept of robustness to explore biomarkers that encode information other than the acoustic one.

In a future study, we plan to validate the results obtained in this study by analyzing more languages and balancing the classes in terms of the number of subjects, age, gender, medication time, PD disease severity, and specific phenotype. It will also be important to balance the number of sentences in the analysis of read speech and compare spontaneous speech samples that report similar speech content. This procedure should be done to avoid external factors that can influence the comparison of the results across languages. In general, it would be ideal for performing an analysis that compares tasks with a similar cognitive load. In addition, results should be commented on considering cognitive impairment (if present). Even though clinical cognitive measures (e.g., MOCA scores) have been collected for NLS, we encourage the creators of new data sets to collect these measurements and conduct a more exhaustive analysis. Finally, we plan to extend the analysis to articulatory and phonatory speech aspects to complement the current study and to assess the robustness of the presented biomarkers in a machine-learning framework performing multi-lingual and cross-lingual classification experiments. In doing this, we aim to develop diagnosis-supporting tools whose performances, in addition to being accurate and language-robust, are interpretable by clinicians.

## Data availability statement

The datasets presented in this article are not readily available because of ethical and privacy restrictions. Requests to access the datasets should be directed to the corresponding author/s.

## Ethics statement

The studies involving human participants were reviewed and approved by Johns Hopkins Medical Institution—Institutional Review Board. The patients/participants provided their written informed consent to participate in this study.

## Author contributions

AF contributed to conceptualization, data pre-processing, biomarker extraction, statistical analysis, GitHub repository management, and article writing. LM-V contributed to conceptualization, data collection, project supervision, and article writing. CM and RS contributed to data collection and manuscript revision. TC contributed to biomarker extraction and manuscript revision. AB, JV, and ND contributed to conceptualization, project supervision, and manuscript revision. All authors contributed to the manuscript revision, read, and approved the submitted version.

## Footnotes

^1^https://ieee-dataport.org/open-access/italian-parkinsons-voice-and-speech (accessed February 4, 2023).

^2^https://sox.sourceforge.net/ (accessed January 26, 2023).

^3^https://pypi.org/project/ffmpeg-normalize/ (accessed January 26, 2023).

^4^https://openai.com/blog/whisper/ (accessed February 4, 2023).

^5^https://github.com/jcvasquezc/DisVoice (accessed January 26, 2023).

^6^https://www.fon.hum.uva.nl/praat/ (accessed January 26, 2023).

^7^https://parselmouth.readthedocs.io/en/stable/ (accessed February 2, 2023).

^8^https://www.google.com/search?q=praat+software&oq=praat+software&aqs=chrome.0.0i512l2j0i22i30l8.4071j0j15&sourceid=chrome&ie=UTF-8 (accessed January 26, 2023).

^9^https://github.com/NeuroLexDiagnostics/DigiPsych_Prosody (accessed January 26, 2023).

^10^https://github.com/wiseman/py-webrtcvad (accessed January 26, 2023).

^11^https://spacy.io/models (accessed January 26, 2023).

^12^https://pytorch.org/audio/main/pipelines.html (accessed January 26, 2023).

^13^https://docs.scipy.org/doc/scipy/reference/generated/scipy.stats.kruskal.html (accessed January 15, 2023).

^14^https://www.statsmodels.org/dev/generated/statsmodels.stats.multitest.fdrcorrection.html (accessed January 26, 2023).

^15^Note that UPDRS was available for CzechPD, GermanPD, and Neurovoz, whereas MDS-UPDRS was available for NLS and GITA. Thus, both UPDRS scores and MDS-UPDRS scores were used in this study. We added specifications in Tables 2, 3 where disease severity statistics are reported.
