# Supplementary material for: Multilingual evaluation of interpretable biomarkers to represent language and speech patterns in Parkinson's disease
Source: Front Neurol. 2023 Mar 2;14:1142642. doi: 10.3389/fneur.2023.1142642 (PMC10017962; doi:10.3389/fneur.2023.1142642)
Supplement: Supplementary file 1 [file Data_Sheet_1.pdf]

Supplementary Material

1 SUPPLEMENTARY TABLES

Table S1. English, Colombian, Italian, and Czech speech transcriptions, with their corresponding English translations reported in italics, from the RP tasks.

Read Passage	English transcription
**Poem**	Do not stand at my grave and weep. I am not there. I do not sleep. I am a thousand winds that blow. I am the diamond glints on snow. I am the sunlight on ripened grain. I am the gentle autumn rain. When you awaken in the morning's hush I am the swift uplifting rush Of quiet birds in circled flight. I am the soft stars that shine at night. Do not stand at my grave and cry. I am not there. I did not die.
**Rainbow passage**	When sunlight strikes raindrops in the air, they act like a prism and form a rainbow. The rainbow is a division of white light into many beautiful colors. These take the shape of a long round arch, with its path high above, and its two ends apparently beyond the horizon. There is, according to legend, a boiling pot of gold at one end. People look but no one ever finds it. When a man looks for something beyond his reach, his friends say he is looking for the pot of gold at the end of the rainbow. Throughout the centuries men have explained the rainbow in various ways. Some have accepted it as a miracle without physical explanation. To the Hebrews it was a token that there would be no more universal floods. The Greeks used to imagine that it was a sign from the gods to foretell war or heavy rain. The Norsemen considered the rainbow as a bridge over which the gods passed from the earth to their home in the sky.
	**Colombian transcription / *English translation***
	Ayer fui al médico. Qué le pasa? Me preguntó. Yo le dije: Ay doctor! Donde pongo el dedo me duele. Tiene la uña rota? Sí. Pues ya sabemos qué es. Deje su cheque a la salida. *Yesterday I went to the doctor. What happened to you? He asked me. I said him: ah doctor! Where I put my finger it pains me. Do you have the nail broken? Yes. Then we now know what is happening. Leave your check at the exit.*
	**Italian transcription / *English translation***
	Il papà, o il babbo come dice il piccolo Dado, era sul letto. Sotto di lui, accanto al lago, sedeva Gigi, detto Ciccio, cocco della mamma e della nonna. Vicino ad un sasso c'era una rosa rosso vivo e lo sciocco, vedendola, la volle per la zia. La zia Lulù cercava zanzare per il suo ramarro, ma dato che era giugno (o luglio non so bene) non ne trovava. Trovò invece una rana che saltando dalla strada finì nel lago con un grande spruzzo. Sai che fifa, la zia! Lo schizzo bagnò il suo completo rosa che divenne giallo come un taxi. Passava di lì un signore cosmopolita di nome Sardanapalo Nabucodonosor che si innamorò della zia e la portò con sé in Afghanistan. *The father, or papa as little Dado says, was on the bed. Below him, next to the lake, sat Gigi, known as Ciccio, his mother's and grandmother's boyfriend. There was one next to a stone bright red rose and the fool, seeing her, wanted her for his aunt. Aunt Lulù was looking for mosquitoes for his lizard, but since it was June (or July I don't know) he couldn't find any. He found instead a frog leaping off the road ended up in the lake with a great spray. You know how scared the aunt! The sketch wet his pink suit, which turned yellow like a taxi. It passed by here a cosmopolitan gentleman named Sardanapalus Nabucodonosor who fell in love with the aunt and took her with him to Afghanistan.*
	**Czech transcription / *English translation***
	Když člověk poprvé vsadí do země sazeničku, chodí se na ní dívat třikrát denně. Tak co, povyrostla už, nebo ne? I tají dech, naklání se nad ní, přitlačí trochu půdu u jejích kořínků, nečechrává jí lístky a vůbec ji obtěžuje různým konáním, které považuje za užitečnou péči. A když se sazenička přesto ujme a roste jako z vody, tu člověk žasne nad tímto divem přírody. Má pocit čehosi jako zázraku a považuje to vůbec za jeden ze svých největších osobních úspěchů. *When a person puts a seedling in the ground for the first time, they go to look at it three times a day. So, has it grown up yet or not? One holds his breath, leans over the seedling, presses a soil near its roots, fluffs leaves and takes care of it by performing various actions that he considers to be useful. Nevertheless, if the seedling grows successfully as if from water, one marvels at this wonder of nature. One feels something like an miracle and considers it to be one of the greatest personal achievements.*

Table S2. Spanish, Colombian, Italian and German transcriptions, with their corresponding English translations reported in italics, from the TDU task.

Sentence #	**Spanish transcription / *English translation***
1	Cuando las barbas de tu vecino veas pelar, pon las tuyas a remojar / *When your neighbor's beard you see peeling, put yours to soak*
2	De la calle vendrá quien de tu casa te echará / *From outside will come that who will kick you out from your house*
3	Cuando el diablo no sabe qué hacer, con el rabo mata moscas / *When the devil does not know what to do, it kills flies with its tail*
4	La petaca blanca es mía / *The white flask is mine*
5	No pidas a quien pidió, ni sirvas a quien sirvió / *Do not beg the one who begged, nor serve the person who served*
6	El que a buen árbol se arrima, buena sombra le cobija / *To the one that comes to a good tree, good shade covers him*
	**Colombian transcription / *English translation***
1	Luisa Rey compra el colchón duro que tanto le gusta / *Luisa Rey buys the hard mattress that she so much likes*
2	Los libros nuevos no caben en la mesa de la oficina / *The new books do not fit in the office desk*
3	Laura sube al tren que pasa / *Laura gets on the passing train*
4	Mi casa tiene tres cuartos / *My house has three rooms*
5	Rosita Niño, que pinta bien, donó sus cuadros ayer / *Rosita Niô, who paints well, donated her paintings yesterday*
6	Omar que vive cerca trajo miel / *Omar who lives nearby brought honey*
	**Italian transcription / *English translation***
1	Oggi è una bella giornata per sciare / *Today is a beautiful day for skiing*
2	Voglio una maglia di lana color ocra / *I want an ocher wool sweater*
3	Il motociclista attraversò una strada stretta di montagna / *The motorcyclist crossed a narrow mountain road*
4	Patrizia ha pranzato a casa di Fabio / *Patrizia had lunch at Fabio's house.*
5	Questo è il tuo cappello? / *Is this your hat?*
6	Dopo vieni a casa? / *Will you come home later?*
7	La televesione funziona? / *Is the television working?*
8	Non posso aiutarti? / *Can't I help you?*
9	Marco non è partito / *Marco left*
10	Il medico non è impegnato / *The doctor is not busy*
	**German transcription / *English translation***
1	Peter und Paul essen gerne Pudding / *Peter and Paul like to eat pudding*
2	Das Fest war sehr gut vorbereitet / *The festival was very well prepared*
3	Seit seiner Hochzeit hat er sich sehr verändert / *He has changed a lot since his marriage*
4	Im Inhaltsverzeichnis steht nichts über Lindenblütentee / *There is nothing in the table of contents about lime blossom tea*
5	Der Kerzenständer viel gemeinsam mit der Blumenvase auf den Plattenspieler / *The candlestick fell together with the flower vase on the record player*

Table S3. Statistics reporting medication state (ON/OFF), time since diagnosis (years), time since medication (hours), microphone type (headset/external), gender PD (%), gender CN (%) age range (years) and presence of mask (YES/NO) during recording session for each data sets.

Data set	Medication state	Time since diagnosis (years)	Time since medication (hours)	Microphone	Gender PD (%)	Gender CN (%)	Age range PD	Age range CN	Mask
NLS	ON (L-Dopa)	5	2-5	Headset	F-40% M-60%	F-64% M-36%	50-79	26-83	YES
Neurovoz	ON (L-Dopa)	7	2-5	Headset	F-38% M-62%	F-56% M-44%	41-88	55-83	NO
GermanPD	ON (L-Dopa)	1-30	1-1.5	Headset	F-46% M-64%	F-50% M-50%	27-84	28-85	NO
CzechPD	OFF	0.5-3.5	–	External condenser	F-0% M-100%	F-0% M-100%	34-83	36-80	NO
GITA	ON (L-Dopa)	1-43	3	External condenser	F-50% M-50%	F-50% M-50%	33-81	31-86	NO
ItalianPVS	ON (L-Dopa)	–	–	External condenser	F-32% M-68%	F-54% M-46%	40-80	60-77	NO
